# Highly efficient CRISPR/Cas9-mediated transgene knockin at the H11 locus in pigs

**DOI:** 10.1038/srep14253

**Published:** 2015-09-18

**Authors:** Jinxue Ruan, Hegang Li, Kui Xu, Tianwen Wu, Jingliang Wei, Rong Zhou, Zhiguo Liu, Yulian Mu, Shulin Yang, Hongsheng Ouyang, Ruby Yanru Chen-Tsai, Kui Li

**Affiliations:** 1 State Key Laboratory of Animal Nutrition & Key Laboratory for Farm Animal Genetic Resources and Utilization of Ministry of Agriculture of China, Institute of Animal Science, Chinese Academy of Agricultural Sciences, Beijing, 100193, PR China; 2 Jilin Provincial Key Laboratory of Animal Embryo Engineering, College of Animal Science, Jilin University, Changchun, 130012, PR China; 3 Qingdao Institute of Animal Science and Veterinary Medicine, Qingdao, 266100, PR China; 4 Applied StemCell, Inc., Menlo Park, CA 94025, USA

## Abstract

Transgenic pigs play an important role in producing higher quality food in agriculture and improving human health when used as animal models for various human diseases in biomedicine. Production of transgenic pigs, however, is a lengthy and inefficient process that hinders research using pig models. Recent applications of the CRISPR/Cas9 system for generating site-specific gene knockout/knockin models, including a knockout pig model, have significantly accelerated the animal model field. However, a knockin pig model containing a site-specific transgene insertion that can be passed on to its offspring remains lacking. Here, we describe for the first time the generation of a site-specific knockin pig model using a combination of CRISPR/Cas9 and somatic cell nuclear transfer. We also report a new genomic “safe harbor” locus, named *pH11*, which enables stable and robust transgene expression. Our results indicate that our CRISPR/Cas9 knockin system allows highly efficient gene insertion at the *pH11* locus of up to 54% using drug selection and 6% without drug selection. We successfully inserted a gene fragment larger than 9 kb at the *pH11* locus using the CRISPR/Cas9 system. Our data also confirm that the gene inserted into the *pH11* locus is highly expressed in cells, embryos and animals.

Pig models are often favored over rodent models due to their clinical relevance and high similarity to human physiology and anatomy^1^,^2^,^3^. Traditionally, transgenic pigs have been produced by integrating a gene sequence or a transgene into the genome in a random manner, in which the transgene can be inserted anywhere in the genome^4^. Random integration of a transgene often leads to unstable phenotypes, gene silencing and unpredictable gene expression, and in some cases, this process is mutagenic. For site-specific gene insertion, homologous recombination (HR) was used in the past to engineer the porcine fibroblasts^5^,^6^,^7^, and the correctly targeted fibroblasts were then used in somatic cell nuclear transfer (SCNT) to generate transgenic pigs. This approach is inefficient due to low HR efficiency, laborious, and time consuming. Transposon system such as Sleeping Beauty has been used in the past few years to generate transgenic pigs with RMCE (recombinase-mediated cassette exchange) acceptor sites inserted at a so-call safe-harbor genomic locus^8^,^9^. These “RMCE-ready” transgenic pigs can then be used to insert a transgene of interest through RMCE. Such transposon method requires pre-engineering of the pig genome to contain RMCE acceptor locus and an extensive characterization of the locus being transcriptionally active, therefore is cumbersome. In addition, the copy number of the insertions cannot be controlled and transgenic founders often contain multiple monomeric insertions which will need further breeding to segregate. As a result, methods that allow one-step, site-specific, and single copy transgene insertion would greatly enhance the generation of optimal transgenic pig models.

Site-specific nucleases such as zinc finger nucleases (ZFNs), transcription activator-like effector nucleases (TALENs) and clustered regularly interspersed short palindromic repeat (CRISPR)/ CRISPR-associated protein 9 (Cas9) have recently been developed for effective targeted gene editing^10^,^11^. Cas9 endonuclease from the *Streptococcus pyogenes* type II CRISPR/Cas system can be engineered to produce targeted genome modification under the guidance of a synthetic single guide RNA (sgRNA) with simple base-pair complementarities with a target genomic DNA sequence^11^. Due to its high efficiency and ease in building constructs, CRISPR/Cas9 has been widely used in humans, mice, rabbits, monkeys and several other species^10^,^12^. CRISPR/Cas9 generates site-specific DNA double-strand breaks (DSBs) that can be repaired by nonhomologous end joining (NHEJ) or homology-directed repair (HDR), through which genes of interest can be brought to the target sites and inserted. In 2014, Zhou and colleagues generated the first CRISPR/Cas9 knockout pig model^13^, and afterwards several other groups also achieved gene knockout in pigs by using CRISPR/Cas9^14^,^15^,^16^. As of now, knockin pigs using CRISPR/Cas9 have never been reported.

Here, we show that site-specific gene knockin can be efficiently achieved using the CRISPR/Cas9 system with transgenes as large as over 9kb. Our method involves generating gene modified primary pig fetal fibroblasts first and then using SCNT to generate transgenic pigs. We also describe the identification of a safe harbor, transcriptionally active locus in the pig genome, the *pH11* locus. A combination of the features of the site-specific targeting of CRISPR/Cas9 and the transcriptionally active *pH11* locus offers an efficient and rapid method for precise gene addition in pigs. In addition, our study confirms that a gene driven by a ubiquitous promoter and inserted into the *pH11* locus can be expressed ubiquitously in pig cells, embryos and transgenic pigs.

## Results

### Identification of the *pH11* locus

An important factor for efficient transgene knockin and expression is the requirement of a “safe harbor” genomic locus that allows gene expression without disrupting internal gene function. Although the *Rosa26* locus has been widely used in mice, humans and pigs, it is not ideal for some gene insertions where the *Rosa26* promoter could interfere with transgene expression^17^,^18^,^19^. An alternative genomic locus, the *Hipp11* (*H11*) locus, was first described by Hippenmeyer *et al.*^20^ and further validated in transgenic mice^21^ and in human stem cells^22^. In mice, the *H11* locus is located within an intergenic region between the *Eif4enif1* and *Drg1* genes, which are mapped close to the centromere of chromosome 11. *In vivo* experiments verified that integration and biallelic expression of the transgenes at the *H11* locus did not interfere with mouse viability or fertility. The same study showed that the *H11* locus displays a high level of global transgene expression and a higher rate of interchromosomal recombination when compared with the *Rosa26* locus in mice. The *H11* locus does not contain any promoter, thus allowing the gene of interest to be expressed under its own promoter, for example, a tissue-specific promoter to specifically express the transgene in that tissue. The *H11* orthologous locus in humans is located on human chromosome 22q12.2, between the *DRG1* and *EIF4ENIF1* genes, approximately 700 bp 3′ to the 3′ UTR of human *EIF4ENIF1*. Transgene expression at the human *H11* (*hH11*) locus in human embryonic stem (hES) and induced pluripotent stem (iPS) cells was proven to be robust and ubiquitous. Given the data from transgenic mice and human stem cells, we reasoned that the *H11* locus would likely support transgene expression in pigs and serve as a “safe harbor” and transcriptionally active genomic locus for transgene insertion.

**Figure 1:**
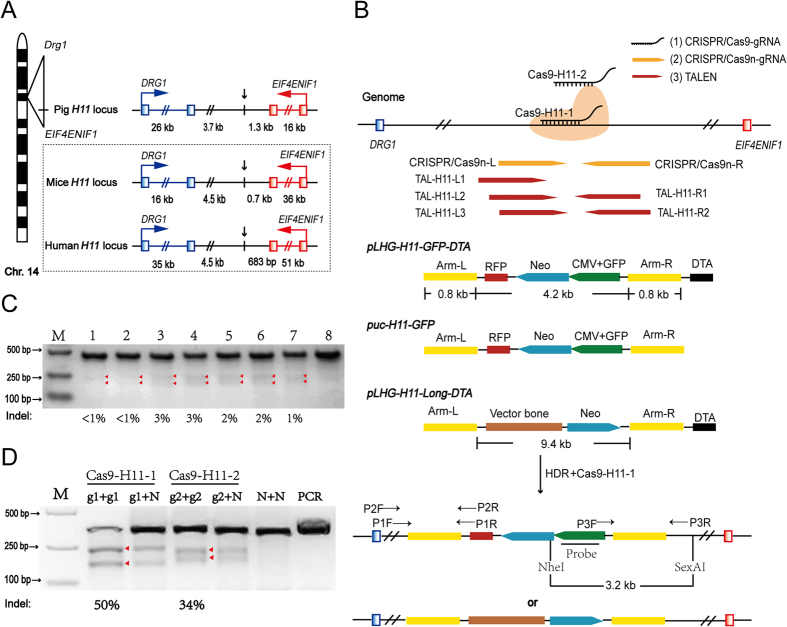
*pH11* locus and knockin strategy. (**A**) The location of the *H11* locus in the pig genome (*pH11*) is in an intergenic region on chromosome 14, flanked by the *Drg1* and *Eif4enif1* genes. Distances from *pH11* to the terminal exons of the two flanking genes are 3.7 kb and 1.3 kb, respectively. The molecular structure of the mouse, human, and pig *H11* loci are highly conserved. (**B**) Scheme for site-specific knockin in pigs via (1) CRISPR/Cas9, (2) CRISPR/Cas9n (nickase), and (3) TALENs. Cas9 gRNAs are in black line (Cas9-H11-g1 and Cas9-H11-g2), Cas9n gRNAs are in dark yellow, and TALEN pairs are indicated in red arrows. Three different donors (*pLHG-H11-GFP-DTA*, *puc-H11-GFP* and *pLHG-H11-Long-DTA*) were integrated into this location using three drug selection schemes, including positive/negative selection, positive selection and no selection. (**C**) Targeting efficiency of TALENs and the CRISPR/Cas9n system at the *pH11* locus. Efficiency was calculated by the ratio of the cut band (248 bp and 199 bp)/uncut band (447bp), and the values are listed below the gel as percentage of indel. Red arrowheads indicate cut DNA bands. M: DNA marker, 1: TAL-H11-L1 + TAL-H11-R1, 2: TAL-H11-L2 + TAL-H11-R1, 3: TAL-H11-L3 + TAL-H11-R1, 4: TAL-H11-L1 + TAL-H11-R2, 5: TAL-H11-L2 + TAL-H11-R2, 6: TAL-H11-L3 + TAL-H11-R2, 7: CRISPR/Cas9n and 8: Negative control. (**D**) Targeting efficiency of Cas9-H11-g1 and Cas9-H11-g2 is 50% and 34%, respectively, by gray analyses. Red arrowheads indicate cut DNA bands. “N” represents DNA isolated from cells without gene editing constructs. Full-length gels are presented in Supplementary Figure S4. The gels have been run under the same experimental conditions.

To identify the *H11* orthologous locus in the pig genome, we first located *DRG1* and *EIF4ENIF1* in pig chromosomes and identified the pig equivalent locus *pH11* by its distance from these two genes. Pig *DRG1* and *EIF4ENIF1* are located on chromosome 14q. The distances between these two genes are very similar among mice, humans and pigs. The intron/exon organization of *DRG1* and *EIF4ENIF1* is also highly conserved among these three species. We screened the region and chose a site where primers could be readily designed for amplification of homologous arms. The *pH11* site we identified is 3.7 kb 3′ of *DRG1* and 1.3 kb 5′ of *EIF4ENIF1* (Fig. 1A).

### Efficiency of CRISPR/Cas9, Cas9n and TALENs

**Figure 2:**
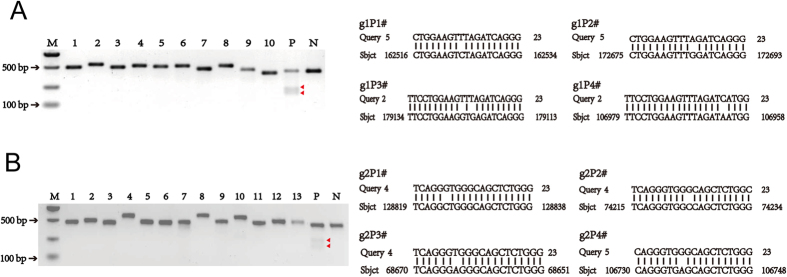
Off-target analysis of the CRISPR/Cas9 system. Sequences on the right are examples of predicted off-target sites for the CRISPR/Cas9 gRNAs (A) Cas9-H11-g1 and (B) Cas9-H11-g2. PCR primers were designed to amplify fragments for each off-target site, and the PCR products were digested by T7EI to detect modified fragments with nucleotide mismatch. The gel pictures on the left represent the T7EI digestion results: none of the predicted off-target PCR products were digested by T7EI, suggesting that no detectable off-target cleavage occurred for the two sgRNAs. M: DNA marker, P: positive control (T7EI digestion of the *H11* locus PCR products amplified from cells transfected with Cas9-H11-g1 (for A) or Cas9-H11-g2 (for B)), red arrowhead indicating cut DNA fragments, N: negative control (T7EI digestion of the *H11* locus PCR products from cells without gene editing constructs). Full-length gels are presented in Supplementary Figure S4. The gels have been run under the same experimental conditions.

Three genome-editing approaches, TALENs, CRISPR/Cas9 and CRISPR/Cas9n (nickase), were investigated to develop an efficient gene knockin system. According to the *pH11* locus sequence, two sgRNAs for the CRISPR/Cas9 system (supplementary S1 Data), six pairs of TALENs (supplementary S2 Data) and one pair of sgRNAs for the CRISPR/Cas9n system (supplementary S3 Data) were designed. The overall strategy is shown in Fig. 1B. The targeting and DNA cutting efficiency by these three gene-editing methods was tested by a T7 endonuclease I (T7EI) assay and confirmed by sequencing. T7EI digestion assays indicated that the targeting/cutting efficiencies of the six pairs of TALENs and one pair of CRISPR/Cas9n system were <3% (Fig. 1C). In contrast, the efficiency of the CRISPR/Cas9 system that used two sgRNAs, Cas9-H11-g1 and Cas9-H11-g2, reached 50% and 34%, respectively (Fig. 1D). When sequencing was used to more precisely detect the gene targeting efficiency, Cas9-H11-g1 reached 64%, whereas Cas9-H1-g2 reached 57% (supplementary S1 Figure). To predict potential off-target activity, 10 and 13 putative off-target sites in the pig genome were identified and analyzed for Cas9-H11-g1 and Cas9-H11-g2, respectively (Fig. 2 and supplementary S1 Data). T7EI assays showed that none of the PCR products of the predicted off-target sites were digested by T7EI, suggesting that off-target cleavage did not appear to occur for the two sgRNAs in all the 23 highly similar sites predicted, at least within the detection scope of T7E1 digestion (Fig. 2). Thus, our CRISPR/Cas9 system is relatively safe for knockin at *pH11*. Cas9-H11-g1, which had the highest activity, was selected to target the *pH11* locus.

### CRISPR/Cas9 mediated site-specific GFP insertion in pig primary fetal fibroblasts

**Figure 3:**
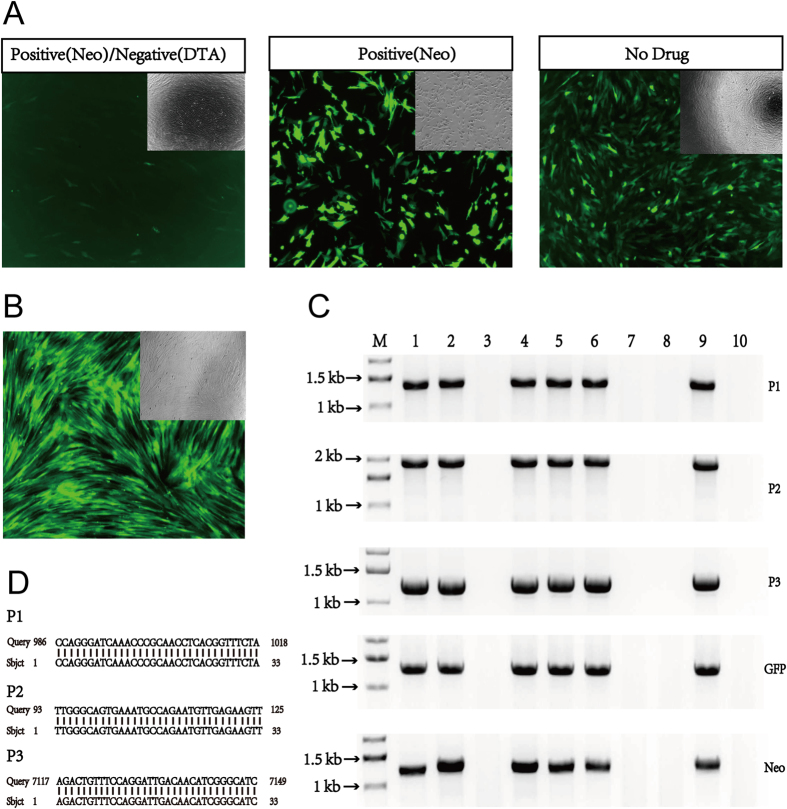
Generation of pig PFF cell lines with site-specific GFP integration. (**A**) Transfected PFF cells 3 days after positive/negative drug selection (left image), positive drug selection (middle image) and no drug selection (right image). The insets show bright-field images. GFP was not detected until after day 8 in positive/negative drug selected cells (data not shown). (**B**) Examples of positive GFP cell clones after clone expansion. The inset shows bright-field image. (**C**) Representative genomic PCR results for screening positive clones. M: DNA marker, # 1, # 2, # 4, # 5, # 6 and # 9 are positive clones and # 3, # 7, # 8 and # 10 are negative clones. P1 and P2 amplified the 5’- junction, whereas P3 amplified the 3’- junction (see Fig. 1B for primer locations). Full-length gels are presented in Supplementary Figure S4. The gels have been run under the same experimental conditions. (**D**) Representative Blast results comparing PCR fragment sequences with genomic junction sequences. See a full Blast comparison in Supplementary Information. Note: Both bright field images and fluorescence images were in 40 × magnification.

We first utilized a positive and negative selection method to insert GFP into the *pH11* locus in pig primary fetal fibroblast (PFF) cell lines derived from Bama mini pigs. The targeting donor vector *pLHG-H11-GFP-DTA* contained a reporter cassette with neomycin resistance (Neo) and green fluorescence protein (GFP) genes driven by the CMV promoter, as well as the red fluorescence protein (RFP) gene without a promoter. The reporter cassette was flanked by a 0.8 kb homologous arm to the *pH11* locus on each side with the diphtheria toxin A (DTA) gene at the 3′ end (Fig. 1B). The promoterless RFP was included to test if any adjacent promoters could initiate ectopic expression. The total size of the fragment between the two homologous arms was 4.2 kb. The donor vector was linearized by BclI digestion and introduced into PFF cells along with the Cas9-H11-g1 and Cas9 vectors by nucleofection (Amaxa). After 3 days of culture, the PFF cells were plated at 10,000-20,000 cells per 10 cm dish. G418 selection for Neomycin started 2 days later and continued for 12 days. Green fluorescence was not detectable until day 8 after selection (Fig. 3A, left) and most clones reached a high level of green fluorescence 10 days after selection. To identify cell clones that had GFP inserted at the *pH11* locus, clones with positive green fluorescence were picked and genotyped using 3 pairs of primers (two for the left arm (H11-P1-F and H11-P1-R, H11-P2-F) and one for the right arm (H11-P2-R, H11-P3-F and H11-P3-R) (Table S2) to amplify the PCR products. The PCR products were then sequenced. All 31 positive clones contained correct recombinants with the transgene inserted specifically at the *pH11* locus (Fig. 3C,D, Figure S2). GFP expression in these pH11-GFP PFF cell clones was further observed by fluorescence stereomicroscopy. Robust green fluorescence was detected in all 31 positive clones (Fig. 3B). No red fluorescence was detected in any of the clones, indicating that adjacent promoters could not initiate expression.

As a delay in GFP expression was observed with the double selection method, we suspected that DTA might adversely affect gene insertion rate; thus, positive screening using only Neo selection was performed in the following experiments. The DTA gene was first removed from the *pLHG-H11-GFP-DTA* vector to create the donor vector *puc-H11-GFP* (Fig. 1B). Three days after nucleofecting PFF cells with the linearized *puc-H11-GFP* donor, Cas9-H11-g1 and Cas9 vectors, the cells were plated at a density of 1,000-2,000 cells per 10 cm dish. Green fluorescence was detected after 3 days of culture in most PFF cells (Fig. 3A, middle image). Cells were then selected by adding G418 2 days later and maintained in culture for 12 days. Clones with site-specifically inserted GFP (pH11-GFP) were analyzed using the 3 pairs of primers described above for the positive and negative selection method (Table S2), and the PCR products were sequenced. All of the 104 positive clones fluoresced green and none fluoresced red (Fig. 3B, middle image and Figure S3).

In view of the high gene insertion efficiency of our CRISPR/Cas9 system, we predicted that the correct pH11-GFP clones could be obtained without any antibiotics/toxin selection. To test this prediction, linearized *puc-H11-GFP* vector was transfected into PFF cells with the Cas9-H11-g1 and Cas9 vectors by nucleofection. After 3 days of culture, green fluorescence was detected in most PFF cells (Fig. 3A, right). Cells were then plated at 50–100 cells per dish for limited dilution. After genotyping and fluorescence observation, 22 clones were identified to be positive for site-specific insertion of GFP (Fig. 3C,D, Figure S2). All of the 22 clones expressed GFP and none expressed RFP.

To investigate whether the system would be suitable for inserting large DNA fragments, the GFP gene was replaced by a 9.4 kb fragment containing the Neo resistance gene and some bacterial backbone DNA, resulting in the donor vector *pLHG-H11-Long-DTA* (Fig. 1B). The linearized donor, Cas9-H11-g1 and Cas9 vectors were then co-transfected into PFF cells, and selection was performed using the positive and negative selection method described above. A total of 23 positive clones with site-specific insertion of the 9.4 kb fragment were obtained and confirmed by genotyping (Table 1).

### CRISPR/Cas9 allows high-efficiency gene insertion in pig PFF cells

Our CRISPR/Cas9 knockin system allowed high efficiency in gene insertion in pig PFF cells. From our PCR and sequencing results (Table 1), a total of 31 positive clones were obtained out of 132 clones using positive and negative selection methods (23% efficiency). Interestingly, the efficiency with the positive selection method was higher at 54% (104 clones out of 192 total clones screened). One possible explanation for this result is that the DTA gene may reduce site-specific insertion efficiency through down-regulation of sgRNA and/or Cas9. DNA fragments as large as 9.4 kb were efficiently inserted at the *pH11* locus. A total of 23 out of 96 clones (24%) carried site-specific insertions of the 9.4 kb fragment from the positive and negative selection method. This efficiency is similar to that of insertion of the smaller 4.2 kb donor. Furthermore, a total of 22 positive clones out of 362 clones (6%) were obtained without any drug selection. All positive clones obtained from this study carried one copy of the inserted gene and they were all hemizygotes. Thus, our CRISPR/cas9 system with the Cas9-H11-g1 gRNA provides an efficient genome-editing tool for site-specific insertion of DNA fragments as large as 9.4 kb.

### Cloning of a pig with GFP expression

**Figure 4:**
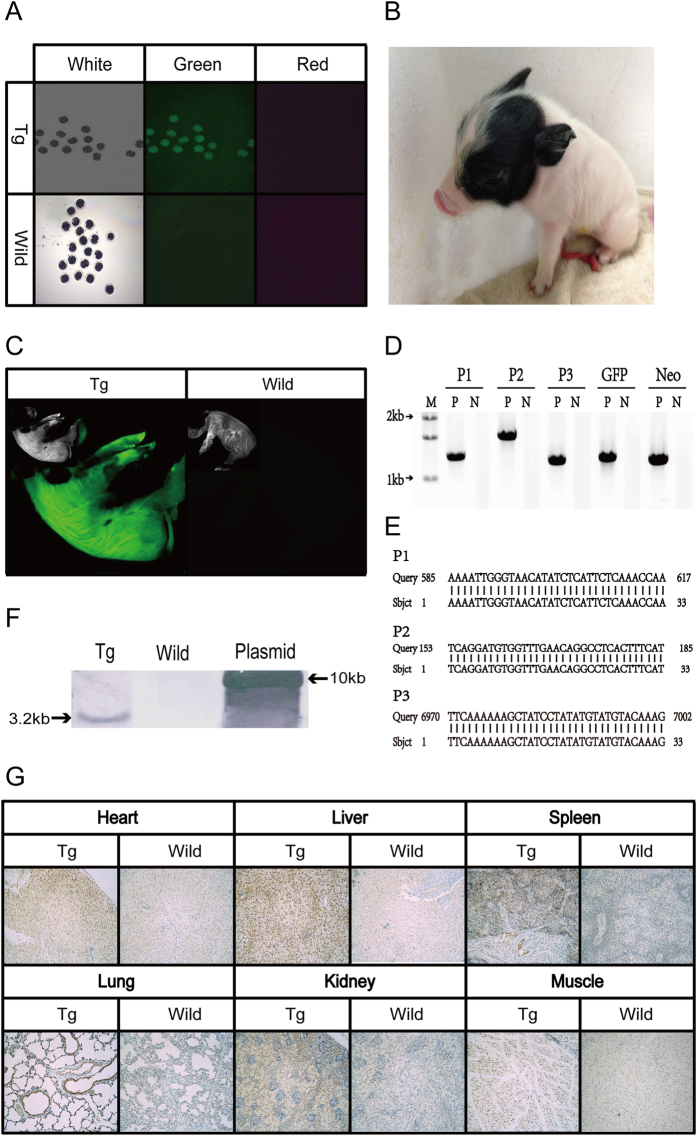
GFP expression in transgenic embryos and piglets. (**A**) Transgenic embryos from SCNT or wildtype embryos at 2-cell and 4-cell stage under a fluorescence stereomicroscope. Left column shows bright-field image. Only the transgenic embryos shows green fluorescence. Red fluorescence is not detected in all embryos. (**B**) An image of the transgenic piglet under white light. (**C**) Images of the transgenic piglet and a wild type piglet under a fluorescence imaging system (Bruker). Insets show images under white light. (**D**) Genomic PCR of the transgenic piglet indicating site-specific GFP gene integration. Primers and PCR products are described in Fig. 3. (**E**) Blast results (partially shown) comparing PCR sequences with genomic junction sequences. (**F**) Southern Blot results confirmed the transgenic piglet contained site-specific insertion of GFP. DNA probe detects a 3.2 kb fragment indicating the site-specific insertion event. (**G**) Immunohistochemistry (IHC) staining of GFP in tissue sections of the transgenic piglet and a control wild type piglet.Tg: Transgenic; Wild: Wild-type. P1, P2 are primers amplifying 5′ junction; P3 are primers amplifying 3′ junction. Full-length blots/gels are presented in Supplementary Figure S4.

A correctly targeted PFF cell line with the 4.2 kb insert obtained from the positive and negative selection method was used as a donor cell line for SCNT. A total of 243 embryos were reconstructed,, activated, and transferred into one surrogate mother. One cloned piglet was born 114 days after the reconstructed embryos were activated (Fig. 4B). To confirm that the cloned pig contained a GFP insertion at the *pH11* locus, genomic DNA was extracted from the pig’s ear tissue and examined by PCR and sequencing. As expected, homology directed recombination occurred at the *pH11* locus with expected 5′ and 3′ PCR junction fragments and sequences (Fig. 4D,E). Site-specific insertion was further confirmed by Southern Blot analysis (Fig. 4F), and a 3.2kb DNA band indicated correct targeting.

To verify whether GFP driven by a CMV promoter at the *pH11* locus can be efficiently expressed in a cloned piglet, GFP expression was examined in embryos, piglets, and representative postnatal tissues. As shown in Fig. 4, GFP was observed in 2-cell and 4-cell stage embryos prior to embryo transfer (Fig. 4A) and in the new-born transgenic piglet (Fig. 4C), and was later present in various tissue sections in the cloned piglet detected by immunochemistry staining. GFP expression was present in the heart, liver, spleen, lung, kidney and muscle tissues (Fig. 4G). GFP expression observed in 2 to 4-cell embryos may have originated from the cytoplasm of the donor cell. This is because the transition from the maternal to the embryonic expression of the genome is after the 4-cell stage in the pig.

## Discussion

In this study, we developed a novel strategy to achieve precise, site-specific gene integration in pigs by combining CRISPR/Cas9-assisted homology directed recombination (HDR) with a newly described *pH11* locus for efficient gene insertion and expression. Using CRISPR/Cas9, gene integration efficiency as high as 50–60% can be achieved. Reporter GFP expression is present in both embryos and animal tissues. Our method offers many advantages over random transgene integration methods, such as no position effect, stability of single-copy insertion, and higher efficiency. This method is also considerably simpler, more rapid and efficient than targeted transgenesis using traditional homologous recombination (HR) in PFF cells or transposon-assisted method. Efficiency in targeted transgenesis using traditional HR is low at <6% with drug selection^6^,^7^. Construction of a traditional HR gene-targeting vector is technically challenging and time consuming due to its large size and many components^10^.

In order to establish an efficient gene knockin system, we started out by investigating three genome-editing approaches, TALENs, CRISPR/Cas9 and CRISPR/Cas9n (nickase). Our data showed that CRISPR/Cas9 has a higher efficiency in targeting and cutting DNA at the pig *H11* locus compared to CRISPR/Cas9n or TALENs. This result was anticipated since CRISPR/Cas9 was shown in other published studies to be more efficient than the TALEN or ZFN system. For the *pH11* locus, the one gRNA for Cas9 turned out to be effective in targeting the locus. On the other hand, the fact that we were only able to obtain heterozygous gene knockin is consistent with the notion that CRISPR/Cas9 is still not efficient for gene knockin. This is mainly because non-homologous end joining (NHEJ) repair over-competes homology-directed repair (HDR) once a DNA double strand break is made by Cas9. Previous studies have shown that the frequency of CRISPR/Cas9-assisted HDR could be improved by adding factors that inhibit NHEJ such as Scr7^23^,^24^. Small molecules that enhance CRISPR-mediated HDR have also been reported^25^. We intend to investigate the effects of these molecules on HDR in our future study.

Our work demonstrated for the first time that CRISPR/Cas9 can be used efficiently in generating knockin pig models. As high as 50-60% targeting efficiency was achieved in pig PFF cells at *pH11* using CRISPR/Cas9-assisted HDR. These numbers are higher than those previously reported using CRISPR/Cas9, suggesting that the *pH11* locus may be in an open chromatin region and serve as a “hot spot” for gene insertion. In addition, our knockin system is effective not only for knocking-in a 4.2kb fragment, but is also suitable for a large fragment of 9.4kb. The fragment size appeared to have no obvious impact on the gene insertion efficiency using this CRISPR/Cas9 system.

We also generated pig PFF knockin cell lines with no drug selection at an efficiency of up to 6%. Although adverse effects from Neo and other drug selections to cells and animals have not been reported, the fact that drug selection may harm the cells or animals in ways that we currently do not understand or cannot detect cannot be dismissed. Additionally, leaving drug selection genes in the genome of genetically modified species when they are used as food is not ideal. Drug gene expression could also interfere with internal gene expression. Thus, in some cases, removing the Neo gene from transgenic pigs at the final step is necessary. Traditionally, the Neo gene is inserted into the genome flanked by two loxP sites and removed by Cre^19^,^26^. Because PFF cell lines cannot sustain two rounds of selection, successive cloning is required to remove the Neo selection marker. Our results demonstrate that the CRISPR/Cas system is efficient at generating “clean” (with no drug marker) knockin pigs in one step, and saves time and cost for safer transgenic animal production. We also learned that DTA selection lowered the efficiency in gene targeting possibly by inhibiting the function of the CRISPR/Cas9 system^27^,^28^.

With the one cloned piglet, we were able to show that the *pH11* locus supports robust, ubiquitous GFP expression driven by the CMV promoter. Further studies with a tissue-specific promoter driving GFP expression are needed in order to verify if the *pH11* is a true “safe harbor” locus. In addition, analysis showing that the nearby gene expression is not affected would further strengthen the notion that *pH11* is a safe locus for gene insertion. It would also be informative to compare the *pH11* locus in supporting gene expression with other known safe harbor locus such as the *Rosa26* locus. In addition, more cloned pigs need to be generated and bred in order to confirm germline transmission.

In summary, genome editing by CRISPR/Cas9-mediated HDR represents a practical strategy for rapid and precise generation of transgenic pigs. Targeted transgene insertion at the transcriptionally active locus *pH11* enables ubiquitous gene expression driven by a ubiquitous promoter such as CMV. Using this knockin system in pig PFF cells with a subsequent cloning step, transgenic pigs with confirmed insertion of any gene of interest can readily be obtained in as short as 6 months without the need of drug selection. We expect that our knockin system can be adopted and used to generate transgenic animals in other large animal species.

## Methods

All experimental protocols were approved by the Institute of Animal Science of the Chinese Academy of Agricultural Sciences.

### TALENs construction

Six pairs of TALENs, composed of three primers on the left 5’ arm and two on the right 3′ arm (Fig. 1B, red), were designed according to the *pH11* locus through online software: https://tale-nt.cac.cornell.edu/. TALENs were constructed using a SIDANSAI TALEN Assembly Kit (1802-030, Shanghai, China).

### CRISPR/Cas9n construction

One pair of sgRNAs targeting the *pH11* locus (Fig. 1B, dark yellow) was designed using online software: http://zifit.partners.org/ZiFiT/Disclaimer.aspx. Oligonucleotides coding for the sgRNAs were annealed and assembled with a pX335 vector (Addgene) using the method described by Zhang at the Broad Institute of MIT.

### CRISPR/Cas9 construction

Two sgRNAs (Fig. 1B, black) were designed according to the *pH11* locus using online software, as described above. The guide sequence were inserted into gRNA expression vector backbone (pU6-gRNA) using annealed oligonucleotides following the instructions of a Cas9/gRNA Construction Kit (ViewSolid Biotech, Beijing, Catalog. No. VK001-01). Plasmid pCAG-t7-Cas9 (ViewSolid Biotech, Beijing) was used as the Cas9 expression vector.

### Targeting efficiency test of the genome editing constructs

PFF cells were transfected with various gene targeting constructs, including six pairs of TALENs, one pair of CRISPR/Cas9n and two CRISPR/Cas9 vectors. For CRISPR co-transfection, the ratio of the Cas9 to gRNA vectors was 1:1 (detailed below), and the total DNA was 5 μg per 10^6^ cells. Transfection was performed using a Nucleofector^TM^ (AMAXA, Cat. No. VPI-1002), according to the manufacturer’s guidelines using program T-016. PFF cells transfected with TALENs were cultured at 30.0 °C for 72 hours, whereas PFF cells transfected with CRISPR/Cas9n or CRISPR/Cas9 were cultured at 37.0 °C for 72 hours for recovery before moving to drug selection. After isolating genomic DNA from the transfected PFF cells, the pH11-up and pH11-dn (Table S2) primers were used to amplify the target region. PCR products were then digested with T7 endonuclease I (T7EI) (Method S1), and targeting efficiencies were tested by gray analyses using Image-J software^29^. To further confirm the efficiency of the CRISPR/Cas9 systems, the PCR products were subcloned into the pMD18-T vector (TaKaRa, Code No. 6011), and up to 40 bacterial colonies each for CRISPR/Cas9 and CRISPR/Cas9n were picked and sequenced.

### Off-target analyses of CRISPR/Cas9

Using two sgRNAs for the CRISPR/Cas9 system and all four possible PAM sequences NGG (AGG, TGG, CGG and GGG) at the 3′ end, a Blast of the pig genome on the NCBI website was performed, identifying the top 10 sites with the highest similarity to Cas9-H11-g1 and the top 13 sites with the highest similarity to Cas9-H11-g2. Twenty-three sets of primers (supplementary S1 Table) were designed to amplify the 23 potential off-target sites from the genomic DNA isolated from the transfected PFF cells. The PCR products were digested with T7 endonuclease I (T7EI) to detect any off-target events.

### Donor vector construction

Detailed donor vector sequences are listed in the Supporting Information (supplementary S4 Data). Both the 5′ and 3′ homologous arms for HDR at the pig *H11* (*pH11*) locus in the *pLHG-H11-GFP-DTA* plasmid were 800 bp. To remove DTA and generate the *puc-H11-GFP* plasmid, the *pLHG-H11-GFP-DTA* plasmid was digested with AscI and FseI to release the *puc-H11-GFP* fragment, which was then ligated to the puc19 vector. The *pLHG-H11-Long-DTA* vector was generated by replacing the fragment containing GFP in the *pLHG-H11-GFP-DTA* vector with a 9.4 kb fragment containing the Neo gene.

### Stable cell line generation

All experimental procedures using animals were conducted in accordance with Administrative Panel on Laboratory Animal Care (APLAC) protocol and the institutional guidelines provided by the Chinese Academy of Agriculture Sciences. PFF cell lines were established using a pig fetus (~day 35) of the Bama miniature pig. The fetal body was disaggregated without its head, bones and viscera and then cultured in Dulbecco’s modified Eagle’s medium (DMEM, GIBCO) supplemented with 15% fetal bovine serum (FBS) at 37.5 °C and 5% CO_2_ in a humidified incubator.

Nucleofection was performed using Nucleofector^TM^, following programs described in the manufacturer’s manual (Amaxa).

For generation of cell lines with GFP integration using the positive and negative selection method, 3 μg of the *pLHG-H11-GFP-DTA* plasmid was linearized by BclI. A total of 1 μg of Cas9-H11-g1 and 1 μg of Cas9 were used for CRISPR/Cas9-mediated recombination. After 72 hours of cell culture, the cells were plated onto 10 cm^2^ dishes (10,000-20,000 cells per dish). Cells were then selected with 0.6 mg/ml G418 (Invitrogen) for HDR. Surviving clones were picked on day 12 after drug selection and expanded for further experiments.

The generation of cell lines with GFP integration using the positive selection method was similar to the procedure described above, except that the donor DNA was 3 μg of the *puc-H11-GFP* vector, and the cells were plated at a density of 1,000-2,000 cells per 10 cm dish.

For no drug selection, PFF cells were transfected with 3 μg of the linearized (by SspI) *puc-H11-GFP* plasmid, 1 μg of the Cas9-H11-g1 and 1 μg of the Cas9 vector. After transfection, limited dilution was used to distribute approximately 50–100 cells per 10 cm^2^ dish. Individual cell clones were isolated in 10–12 days and expanded. Genomic DNA was isolated from each clone, the PCR results were verified and the sequences were analyzed. Positive clones were cryopreserved after a total of 15–18 days in culture.

Transfection of the *pLHG-H11-Long-DTA* vector containing the large 9.4 kb fragment was performed using the same transfection procedure described for the positive and negative selection method above.

### Genomic PCR detection

To test cell clones and animals for site-specific or random insertions, we performed five PCR reactions: the 5’- junction, 3’- junction, Neo gene, GFP gene and *pH11* genomic locus. To make our test more convenient, two pairs of universal primers were designed for the 5’- junction and one pair for the 3’- junction. All of these primers are listed in Table S2.

### Southern Blot detection

To confirm transgene insertion in the pig genome, Southern Blot was performed using DIG High Prime DNA Labeling and Detection Starter Kit II (Roche, 11585614910). DNA was isolate from the transgenic (Tg) piglet and a wild type Bama pig tissue, and digested by NheI and SexAI. Plasmid *pLHG-H11-GFP-DTA* was used as a positive control. The primers (Southern-F and Southern-R) used for the DNA probe amplification are listed in Table S2 and the probe hybridizes to a 3.2kb fragment depicted in Fig. 1B (between NheI and SexAI), indicating site-specific gene insertion.

### Somatic cell nuclear transfer

All experimental procedures using animals were conducted in accordance to APLAC protocol and the institutional guidelines provided by the Chinese Academy of Agriculture Sciences. The cloned pig was generated by SCNT (Method S1). Briefly, nuclei of a GFP positive cell line selected by positive and negative selection were microinjected into enucleated pig oocytes. The reconstructed embryos were activated and cultured to develop into blastocysts. High quality blastocysts were transferred into a synchronized pseudo-recipient female pig to carry the embryos to term.

### Fluorescence detection

GFP positive cell clones and reconstructed embryos were viewed under a fluorescence stereomicroscope (Nikon). The transgenic piglet and a wild type Bama pig were both euthanized on one day after birth and observed under a fluorescence imaging system (Bruker). Multiple tissues from the two piglets, including the heart, liver, spleen, lung, kidney and muscle were harvested, fixed in 4% paraformaldehyde in phosphate buffer (pH 7.4), embedded in paraffin, sectioned, immune-stained with GFP antibody (Santa Cruz biotechnology, sc-8334), and observed under a light microscope (Olympus).

## Additional Information

**How to cite this article**: Ruan, J. *et al.* Highly efficient CRISPR/Cas9-mediated transgene knockin at the H11 locus in pigs. *Sci. Rep.* **5**, 14253; doi: 10.1038/srep14253 (2015).

## Supplementary Material

Supplementary Information

## Figures and Tables

**Table 1:** Summary of HDR experiments for generating cell lines with site-specific gene insertion.

Selection Method	DNA Size (kb)	Total Clone Numbers	Positive Clone Numbers	Positive Rates
Positive/Negative	4.2	132	31	23%
Positive	4.2	192	104	54%
No Dug	4.2	362	22	6%
Large Fragment	9.4	96	23	24%

The “large fragment” used the positive and negative selection method.
