# Supplementary material for: Highly efficient CRISPR/Cas9-mediated transgene knockin at the H11 locus in pigs
Source: Sci Rep. 2015 Sep 18;5:14253. doi: 10.1038/srep14253 (PMC4585612; doi:10.1038/srep14253)
Supplement: Supplementary Information [file srep14253-s1.doc]

**Supplementary Information**

**Title:**

Highly efficient CRISPR/Cas9-mediated transgene knockin at the H11 locus in pigs

**Author list:**

Jinxue Ruan, Hegang Li, Kui Xu Tianwen Wu, Jingliang Wei, Rong Zhou, Zhiguo Liu, Yulian Mu, Shulin Yang, Hongsheng Ouyang, Ruby Yanru Chen-Tsai, Kui Li

**Method S1**

***Mutation detection using T7 Endonuclease I assay***

The T7 endonuclease I (T7EI) assay is used to measure targeting efficiency of the CRISPR/Cas9 system. T7EI cleaves with high specificity at the 3′ side of any mismatch site in both DNA strands, including all base substitutions and insertion/deletions up to at least 12 nucleotides. In the CRISPR/Cas9 system, the gRNA targets specific genomic locus and Cas9 makes the double-strand DNA breaks. The cellular repair mechanism then repairs the DNA breaks predominantly through the non-homologous end joining (NHEJ) pathway, creating nucleotide insertions or deletions (indels). These mutations can then be detected and quantified by T7EI assay. The T7EI assay involves four steps: (1) PCR to amplify target DNA from both mutant and wild-type reference DNA; (2) hybridization to form heteroduplexes between mutant and wild-type reference DNA; (3) treatment of annealed DNA with T7EI nuclease to cleave heteroduplexes; and (4) analysis of digested DNA products using gel electrophoresis. For our studies, 200 ng of purified PCR product was denatured and re-annealed in NE Buffer 2 (New England Biolabs) using a thermocycler to first heat the DNA at 95 °C, and then cool to room temperature. Hybridized heteroduplexes were treated with 0.5μl of T7 Endonuclease I at 37 °C for 30 min in a reaction volume of 10.5 μl. The resulting products were electrophoresed in 2% gel and T7EI cut efficiency were calculated by the ratio of cut band vs. non-cut band. .

***Somatic cell nuclear transfer***

The cloned pig was generated by somatic cell nuclear transfer (SCNT) Briefly, cumulus-oocyte complexes (COCs) were aspirated from follicles, maturated for 40h in maturation medium. Mature oocytes were enucleated and fused with the donor cells enriched in G0 of the cell cycle. The reconstructed embryos were activated in medium and cultured to form blastocysts at day 7. High quality blastocysts were transferred into the synchronized recipient pigs 1 day after estrus (about 200 blastocysts / recipient).

**Data S1**

***H11* locus and target site for sgRNA Cas9-H11-g1 (target site in red font)**

GATGGATTGGGATTCAGGTCTCTGCTGTTGTTACTTGAGATTGCTTTCTAGATTCTACTTGTGGAAACAAAAAGCCTTTGCGAGAATTCTAAACTGGAGTATTTCTGTAATTGAGGAGTCTTGCTCAGCAAATCCCACTTAGGGGACTAATGAAGTACCAGGAAGAGACAGACCATGCTCAATCCACAAAGCCAGGTTTTACTGAAATGTGACCTACTTTCTTATGTTCCTGGAAGTTTAGATCAGGGTGGGCAGCTCTGGGTTTTATAGGCTACACTGTTAACACTCAGGCTGTTTTCTACCGTTTAGTCAAAATATAGTCACCTTGCCTGCTTCACCTGTCCATCAGAGAATGGCCTCATTAATTGACTCTCTAGTATGAAGTCAAAGTAGCTTTGGTGGCCCTAAATGGACAAGTATCAAGAGACTGGGTGAATTGAGGAGCTT

**10 predicted off-target sites for Cas9-H11-g1 in pig genome (off-target sites in red font)**

g1P1

AAAGGAGGCGGAAGGGTAGTACCTGTATGAGGAAAAGCTGGTTCTTCCCCCAGCATTTCCAGATACTTCTCCTTAGGTCTCGTTGATAGAGTGGTGCCTTTAGCCATCTAAAGAAGGCTAAATAGTATTCTAGCTGGGCATCATTGCTCAAAGTGAGGTTGGAGGTCTGTAAATAAGAAGGCAGGAAAAGCAGGCCAGGAGTAGGACGTGACACACTTGGATACACCTTTTCTTAGTCTGCTCTGAGTACTATAACAAATTCCCATAGACCAGGTGGCTTCTAAGCAACAGAAATTTATTCCCCATAGTTCCAGAGGCTGGAAGTCTAGATCAGGGTGCCAAAAAGTTCGCTAAGTGCCTTCTTCCAGGTAACAGACTGATGACCTCTCATTGGTCCTCATGTGAGAGAGACCAAAGGGCAGCAACTTCTCTCCTAACTTTTCTTAAAAGGGCACTGATCCCATTCATGAGGGCTCCACCTTCACAGCTTCCTC

g1P2

GGGCAGCTAAAGAAATTGTGCATGTTCCCATAGCTAGTGGACTGAAAAGCAGGGGTTTAAACCAAGGTAGTCTGGACCCAGAGATCATGTTTTAACCAATTCTGCTGAACTGCCTACGTGCCATTGCTTCATGCGCCTGCTTTCAGGAATCCCAATAGAGTAAAAATTTCTAAAAACCTACACCTACTTTCGAGGGCTCTAAGGACAGTAAGACTCCACACAGGAACATTTCTCAGTGTTTTAGTTGATATGGGATGCTATAACAAAATAGCATCCACTAGGTGGCTTAACTATCAGAAATGAATTCTCACAGTTCTGGAAGCTGGAAGTTTGGATCAGGGTGCCAGTGTGCTTGGTGTCTGGTGAGGATCCTCTTCTGGCCCGGCAGAAAGAGAGAGGGCATCCGCTTCTTATAAAGACATTCCATCATGCGGGCTCCACCCTCATGGCCCAATTACCTCTCAAAATGCTCACCTCCAAATTCCATCCTCCTGTGGGTTAGGGTTTCAGCATATGAGTTTGAGGGGACACACATGCAGTC

g1P3

GCAACGCGGGATCTAAACTGAATCTGTGACCTGCACCAGCCTACACCACAGCTCATGGCAACGCTGGATCCTTAACCCACTGAGCATGGGATTGAACCTGCATCCTCATGGGTACTAGTCAGGTTCATTAACCGTTGAACCATACAGGAACTCCAGGATGTGGAGAACTTTTGGCTTAGAGGAATGAGAGTTCTGGGATAGAACTCAACTTTAATTATAGTATTCCCTGATCTCACCTTCCAGGAAAATGCCTTCGTGGGGCCAGAAGAAAAGAGGGTTGACACTGAGTTCCTACAGTCCAGTATCTGTGTTCACAGCCTTCACTATCTACTTAATGTACATGGTTTTATTTAATTTCTAAAAAGCATGTCGCTATTTTGCAGAAAAGGAAACTCAGGCTCAAGAAAGATAATTAGGTAATAGTGCTGAAATTTCAAATAGGACTGTATATACAAGAATACTAAGCAGAACATGTTGTCGTGTCTGCTAAGAGGTT

g1P4

CCAGATTAGAGGCAAGTTCGGAGACTTACTTTCCTATTCGCAAGTCCTCATTCTGCAGAGATACTGGGCAGGAATGCCAAGGGGACTCACCATCGTTCAAGCCTGCTTGCAAATTTGCATGAAAAGTATTAACATAACCACTGCAGGCCTGGGCCCCTGGAATAGCCAATGGGCATCTTCAGTGAGAGGAAGGCCATTATCTAAACTTCCAGGAATCCTACTGCAAACCTTTACAGAAAGACCCAAACTCACATTGGATCGGGCTTTTGTGGGTCTCTGGGGAATGCTGACGTTGTTTGTTAATGCCACAACAAATGTGTCCCTGAGTGAGGCGATCGACAGGACACCTGTCTCTGCATTGAAACCTGATTAAAAGCAAGTCCGGCCCCTGCAGTTCTCTCAGGAGTCTTCACAGTTTCTAAGCTGTTTAGTGCTTTGGGATTCAATCAATTCTATGGCAGGAAGACTTCATCCTTGTTTCTTCTAGTGATAATCCATCCACAGCCCAGCATA

g1P5

CTTCATAGCAATTTCCTTATCCATTAAATGGTTAAAATCATCATTTTACAGATTAGGAAACTAAAACTTAGCCAGCCACATAACTTGAAAGTAGTAGAGTTTTAATTTAGGTTTGAATTTAGGCTTGTATAACTTTAGAATCCATGTTCACTAATCATTGGCGATAGGAACTAGCAAATAGATAGGCATTCACTATGCATAGAATCTCGTCATGAGGAAGCATGAGACAAACTCAAATTGACATTCTATAAAATAACTAGCTTATATTTTTCAAAAATGTTAGGATCAGGAAACATAAGAAAGGAGTTCCTGGAAGTTTCCATCATGGCTCAGAGGTAAGGAGCCTGACTAGTATCCATGAGGATGTAGGTACGATCCCTGGCCTTGCTCAGTGAGTTAAGGATCCAGCATTGTTGTGAGCTGTGGTGTAGGTTGTGGAGGTGACTCGGATCCCTTGGCTGTGGTGTAGGCCAGCAGCTACAGCTCCAATTCTACCCTAACCCT

g1P6

AGCAACATTACATTAGGAAACCGAGAAACTAGTTTATTTTTAGTCTACTTTCAAGGTCACAGTTGAATCCCTAGTATATAATCATCATGTAGAAACCATAGGGTACAGAAAGAATTTATTCGCTTCTTAGCCATCCGCTTCACAGAAAAGGAATTCTACAAAAGCTGAACTTGAGAACATGTAAGTTTAGATCATGGAAATATATGCATTATGTTAATCTTTTCATTTTTATATTTTTGGCTTTAATTTGTGATTAGAGAATACCTTTAAGGAAATCATAGCCCATTTACAGACTTTCAATAGTTCTATATTCTGGTTATATTTTTTGTTTCTTTTGGAGGGAATCCAAATCCATAGGGGCCCATTTGATATAGGTTTTCCTAATTTGGAGGCATAAACAAACAAGTGCAAATAGTCATAATAAATAATGTTGGCCAATATTCACAATCTTTCAATTTAATAGTGTAGACATGTTGATAAAAATTCACAGTCCTAAGTTACCACAAGTCA

g1P7

TCCAGACCTTAACTCTCCTGAACTTTCAGTTCAGATATATTTCCCCTTCTCTTTGGCTTCCCATGAGTACAACTATACTTTAGCACTTACCATCATTTAAAGCTCCAATTTCTAAATACAATTTACTTACCGTATGCCTCTCCCAATCCATCACCCGCTGAATATGATATTTGCCATGATCAAAACTTCCAATATGTGGTCACCAAATATTATCTGACGCAATCAGCAATATTCCCTTTCTCTTCTTCAGCACATTTAAAAAATGTGACAACAATAATACAAAAGCTCCTCATCACTGAGCATATTATATACTTTGCAAAAATATGCTACAAACATTTAATTCTCACAGTAATTTGGTGGATTACATTATTATTATTACGTATATTTTACACCTGAGTCAACTAGGCTTAGACATGTAAAGCAACTTGTTTACTTATACATATTTATCAAGTGGCAGGACCAGGAC

g1P8

AGCTGTGTTCAGGTCTCGTCCTATTCAAAAGAGGAAGCACTTCTGTTTTTTTGTTTGTTTGTTTGTTTGTTTTGTCTTTTTGCCATTGCTTGGGCTGCTCCCACGGCATATGGAGGTTCCCAGGCTAGGGGTCCAATCGGAGCTGTAGCCGCCAGCCTATGCCAGAGCCACAGCAACACCAGATCCAAGCCACGTCTGCAATCTACACACAGCTCACGGCAACGCCAGATCCTTAACCCACTGGGCGAGGACAGGGATCAAACTGGCAACCTCATGGTTCCTAGTCAGATTCGTTAACCACTGAGCCATGACGGGAACTCCCCGGAAGCACTTTTCAAGTACAACTATACTGAGAGGAAAACGCTGATTGAAAGCCTCATTTTCTGATTGGCAGTTTAGATCATGGCAGAAAAACCACTCGCCAGATTAATGTGACAACCAGCCAGTCACAAAATCAACTTTTTGTTCCTTTTCTTTTTAGGGCCACACCTAATGGCATGTGGAGGTTCCCAAGCATAGGGGTC

g1P9

AAAAGTTGTATTTCTTCCCTCTCAAATTTCTGGAATTTTTCATATATTTTATTTTGCTTTCTTGTCTTACTGCATTACTTAGACTTTCAGCAAGGTGATAGATAGGAGTGAATAGAAGTGTGATTTGGAGCAATTATATATTTACTGACAAAGTAAATAAATAACTAAACCTTGATCTAAACTTCACAAAATATAAGAAAATTAACTTAGAATGGATTTTATATTTAAATGAAGAATGTACAACTATAAAAACATTAGTGCAATAATAAATATAGAAGAACTATTAATACAAAAAAGCACCTGGCTGAATTTAATATTTACAAAATTATACGAAGTTAAAAAAATGGTAAGTCAAAGTTTACATGTGTAATTTCATTTATATAACTTTTCTGAAATGACATTTTAGAAATTAAAGACAAATTATTGTTACAAAGAACCAGAGAAAGTGATGGGGGAG

g1P10

AAAACCCATGTGACAACCAAGAAAGGTCAACTTGTCCATCCTTCTGGCTCTGAACAGGGCTGCAGGTCAGTCTTATTAGGAACCCTACAGTGAGCTGTGGTTTTTAGTCTTTAGAGATGATTTCCACAACCACCTATGGAAATTCTGTCCATGTCTCATAATTCCATCCGCAAGTTTTCTTAATATTAGACCTACATTGTTCAATTTAACTGCACTATGACTTGGATCAGCTCCGCAAAGTTTAGATCAAGGGTTGCAAACTGGCAGACTGTAGGTTGAAACCAGCCTACAGACATATTTTGTTTTGCCTGCACTGACAAAAATGTTTCAATAGTTGCCAACATTTAAAAATAGGGATAGTCATATTAAAAATCCAGCTCTTGTTGAAATAAATCCTCGGAAAGGATTTGCTTATA

***H11* locus and target site for sgRNA Cas9-H11-g2 (target site in red font)**

GATGGATTGGGATTCAGGTCTCTGCTGTTGTTACTTGAGATTGCTTTCTAGATTCTACTTGTGGAAACAAAAAGCCTTTGCGAGAATTCTAAACTGGAGTATTTCTGTAATTGAGGAGTCTTGCTCAGCAAATCCCACTTAGGGGACTAATGAAGTACCAGGAAGAGACAGACCATGCTCAATCCACAAAGCCAGGTTTTACTGAAATGTGACCTACTTTCTTATGTTCCTGGAAGTTTAGATCAGGGTGGGCAGCTCTGGGTTTTATAGGCTACACTGTTAACACTCAGGCTGTTTTCTACCGTTTAGTCAAAATATAGTCACCTTGCCTGCTTCACCTGTCCATCAGAGAATGGCCTCATTAATTGACTCTCTAGTATGAAGTCAAAGTAGCTTTGGTGGCCCTAAATGGACAAGTATCAAGAGACTGGGTGAATTGAGGAGCTT

**13 predicted off-target sites for Cas9-H11-g2 in pig genome (off-target sites in red font)**

g2P1

AGGACAGCAGTGGTGGGATGGCAGAGGGCCCAGCCGCCCCTGTTCTCGCTCAGACCCTGGAGTGGCTGTGATTGTTTTCTGTTCGGAAACCCACCCCGAATGCAGCTGGGGACCAGAGGTCCAGGCTCCCAGGCCTGAGACAGTTTCCCTGGCCCTCAGGCTGGGCAGCTCTGGGCCCCTCCTGCTCCTTCACCCCTTCTGTCAGGCCAGGCTTCTAATAGGTGGAGAGCTGAGCCAGAGCCGAGGAGGAAGGGGGCTGGGAAGCCCAGAGGACATGCCCAGGAGCAGCAGAAGGGAGCTCTGCATCCAGCACCCCGGTCCCACTGGTGGGATGCGGTGGCCTCTCAACTCCTCTTTCCTCCATGCACCGGCTTCCTCTACTCAGCAGTCTTCCCACCTGCTCTCACATCCACTCCTGCACCTGCACGCATGCATGCGGCTCACAGACTCACACTT

g2P2

AGGATGGACAGGTGTGTTTGGTGCCAGCCTCTGCTGTGGTGAGAATGCGGGATGTGCCAGGGAAATGTGGGCTGACCGCGGACACACAGGCCCGGCTGCAGTACCACCTGGCTGCTGTGCTGAGCCCTCAGGTCCCTCACAGTATTAACGGAAACAGGCTTCTGAAGATGAAGGGATTGTTCTGTGATTTCCTGCTGCTGAGAATAATAAATGTCTTGTTACAAACAAATGTCATGACAGTTACTCTTAGGTGCCATGGATTGATGTCAGGGTGGCCAGCTCTGGGCTACACCACCCACCTCCAGTTTGTACAACAGTGTTGATATATAGGGCTACACTCATTAATGTTCAAGTCTTCTGTGTTGAAAGTTGTGTTTAATTTCTAAAGTTTAAAAAAAGCAAAAAAAATGGTGCTAAACTTTCACCCCTGAGCACGCTCAGTGAGACTGGTCATGCAAGCATTTACAGTGCCATGCTCTTTCAAGCC

g2P3

CCTGCTTAGGTTCCATTTGCCTTTTCACTGGAGTCAGTAAAACTGCCTGGAGACAGCCCAGGAAATCTGGGTGGGCTGGGTTACACAGTTCAACCAGGCAGAGGCCCTGACCACAGCTAGGCGTGGTGGGAGGCAGACGCCTTGACCTGACTGCCCCCAGGGGGCCGCCTGCAGACTGACAAGGGGAGGGTGGCCCAGAGCTGCCCTCCCTGAAGAACTGCAAACGGCTGCCCTAGAGGTGGCGCCCAGTGGTCCTGGGGAGCCACTGGAAACAAGGCCCTCCCTCACTTTACTAAAGCACCCAGGGGCCTGGAACACAGAGGCCACGGAGAGGCCAAGGCCAGAGGGTGGCGCTCCCGTCCCCGAGACAAAGCAATTGCCGGAGCCGGTCCAGGGGACAGCCAGGATCTGGAGGGCCAGAATCTGGAGCCACCATCATGTATGCC

g2P4

CACCAAGTGGAAGTGGGCTCATTTCTGTTCCCAGCAGCTCAGTACTTCTAACGGCCCCCTCCTGGCTCGCCAAGAGGCCACAGCGCCCTACCAAGAGCTCGCTGCCTGGCCTGCGCCCGGCCTTGGGGGTTCCAGGATCTGCTCACACTGCCCCGCTGTCTGCCTCTCAGCCCAGGCGCCGCGGCCTCTACGCCTGCACTAGGGCTCACACAGCCGTGAACCCGCACACACACCCAGGGTGAGCAGCTCTGGGCCAGCACCCCCCATCCCTACAAGCACACTGGTGCATTTCCAGAACAGCAGGCCCCCTTCCCAGCTCAGGTGCCTGGCTGCCTGGGAGAGGGCCGTGGACAAGAGCAAAGCCCCGGGGGAGGCCCAGCACATGCGGTGTTGTTCATACGCCTGACTTCCAGAGGCCCCTCCCTCGGGACTTCTGTGAAAATCAGATCCACATTTGCACAGTTACAAAGCTGGTCCCAAGCCAGGAGGGATTTGGATGTCCTCTAAGGAGCAGGTATTATAAGAGCCTTAGTCTGAAGACGCAGCAATCCAACA

g2P5

ATCCTGGATGTGAGCATTGTGTAAGCTGTAAAATGCTCTAGTTTTTCAATACAACACACTTGTTCTTTCTAGACTGTGCACACCGGAACCCCTATGTCTTTTATAGAAAGTGCCATTTCCAGTGATTCTTATTTACCCCTCAGTGCCAAGGAGGGCTTGAATCACCCTATTGCAAGGTTATCGCATTTTGGCATCTCATAAAATTTTCGCAAGGAAAACTTGCTGCCCTCTCTCTCTGTCTGAAGATGCAAAGGGAAGTGATTAGCAGAACACAGGGTGGGCAGCTTTGGGCAGGGGCTGCCTGATGGGAGTTTTGTGTGGCGGGACCCAGCCAACCACAGCAATTTGGTGGGGAACACACACTGACCTCTTCTCCTTGGCCCTCTGACCTATGGTGACCCTCTTTTCTGGGCTGAACCTAATCCAAAGCCAGACAACAAGGGAGACTGGTTGATGTCTGTCGGCTCC

g2P6

GGTGGGTTAGTCATTTCAGGTGATGGATGCCATCCTGGTGGGATGAGAGTTTAGTTGTGGGGAAGAGTGGGGGGCCTGCGCGTCTGGGGGGACTTGGTAGAGGGACGCCCCTCAGCGTGCTGCCCTCCCCACTGCTCCCGCACTGTGGCTCCCAGATCTCAGCTGGATTGGTGGCAGCTTCTGGTGGCAGCTGGATTGGTGGCAGCTTCTGGTGCCCTAGAGCTGCACACCCTGACACAACGGGGAGAGAAAGAGAGGTGACCTCTGTTAGATTTCAGGAGCTTCCGGGGGAGATTTCAGTCAGGGCCTACAGTGCCCCTTGTGAATGCCTCTGAAGGCGAGTGCCACTGTCCTTGTGGGTGACAGAAGGAGGAAGGACCAAAGGACCTCTGTTAGAGGTGGTAGCATTTCTACCCTTGTTTCTGGCTGGGAAATGCTCGTCCAGCCTTTACTGCGGGTTTCC

g2P7

CACTGCACAGAGGACTTATCACACTGTGGTATAATTGGCTGACTTCCCCATAGACTAGATACCAACCCCCCTACCCCGCCAGTAGACGGAGGGCAGAGGCCATGACTTTATCTGCTTTATACCCCAGGTGCCTCGCTCAGCAAATGCTCATCCTCGATTGAGCCCTTTACTCTGCCAGTCAAATGTGAAATAAGCCCTAGAGCTGCTCACCCTGGGCTACTCACTCAATCATTAGGTCTTTGTGGCTGGCCAACATCTGAGAGAATAATTAGCAACTTGTTTGTGCAACATTTCTGCCTAGTAATCCATCCTCAAACTAATTAAAACAAGGGCTTCTGACTTGATGAATGCTTTGAATACATGAAGGAATATTTTCTAATCCTTCTTATTAAAAAAAAAACTCCTCTAAGTCAATCTAAGGAAATATAAATCTTTTTCTTATTAAATAAGGCCGCCATCAAGGTAAAATC

g2P8

GACAGGTGTAGAGGGAGGCGGCCTGGCTGAGTCGAGGAGAAAGGATATGGAGGTGCAGGTAGGTGGTGAGTGTGCACGTGTGTGCGTGTGCATGAGTGTGTGTGGGGGGAGTCGGTGGCCATTCTCCTCGGAGAGACCGGAAGGGCTGTCATCAACCAAGGTGAGAAGGGAGTGGGTGTGGGTAGGACCCTGCAAATGCAGGAGCAGGGGGGGCAGCTCTAGGAATGTACCTCCCGCGGGTGTATTCTCCTCCTGGGTGGGTGTGCGCATGGAGTAGCAGAGGCATGAATCACTGGTCAGTCATACAAGATCCGTTAGGCAAACATTAAAAAGAAAACTAAAGTGTAAAAAGACGCTCAATGAGGTTCCTCTGTAGCCGTTTCCTCTTTAAAACTTTAAGAAAGGAGGGGCACTGTCTTTAAGGCCTGAGCCTGCGGTGGCCCCCTTTACCCGGCACAGCAACAAAGCTATCTTTTCCTCCTTTAAAATAACTTTAAGAAAGGAGTGGATATGGAGTGCCCATTGCATGCATCAGAAATGAATCTGACTAGTATCCATGAGGATGCTGCTTCCATCCCTGCCTC

g2P9

TTATCCTGGGCTCCTTTCTCTCATGTGCTCCAAAGACCAAAATGCTTCAGATCCACTGTTTTTGTTTCTGGTGCATTAAAACTATTTCCTTTTCTTTCCCCCACCCTTTTTTTCTTTCTGCTTTTCCTGGATCCTGGAGGCTGATGAATACCCTACTTTGCTTTTTAAGTACTTGTTACTTGAATAAAGCTGTGAGGCAGGGTGGGCAGGTCTTGGTTTTGGATGTTAGACTGAGAAGGCCCAAATCTGCCTTCTGTTGTTTATTTAGCAGAGAATTGGGGCTCCTCTAATCTGATATCTTAGTATGATGCTTTCAAATTTGCATTTCCTCAGGGCCTTTGAAGATTTAAAGCCAAATAATTAAGTGGTAGGGAGCTCTGAAAATTGGCAGAGATCAATATTTCAATACCATGGAACCTCTGATACCACTATATTTCACCGTATAGGCAAGAGTATATTATACTCACACAAAATGCAAGCCTG

g2p10

CGAGGATTTTCAGCCCCAGAACAACCTGGAAGAAAAAAGAAATCAGGGGAGCAAGGAAAACCTACTAGACGGGAACACAGTGCTTTGTAGCAAGCTGAGTCCTGCTTTGTCCCTCAGTATCCACTTCCGGTGCTGGGACCATGCTCTGGAAGGCTGGCAGAGGGGCCGGGCTCCCAGGGTTGATTCGTGGGCTCTGTCCCAGAGCTGCCCATTCAACCTGGGCCTGCCTGTCTTCTCTCTCCAGGCCTCCAAGAGCCTCCCATCCCTAGGTCTGCTCCTGGCCCCAAGAGGTGCCCACCCAGATCTGCTTACCCAGGGTCTGCAAAGGATGCCAATGGTTTCCTCACGGGCACCATCCTGGCCTGGCTTCCTGCCCTAAAGCCCTAGGAGACACTTATCTCAGAATGCAGGCCTGAAAGGACAGATGACAAATCAGATGCTAGCCACTCCCAACTCTTCATGGACACCCCCGAGCACCCCTACTCCCTGGGAGTAGCTGTGGAGAGAGAAAAACACATCTACAGGAAGAGCTGGTGAACGGGCACAGATGGGAC

g2P11

ATGGCGACATGGTTAGAGCACATGATGGAAGAAATGTGTCAAATGATAAAATCTGTGTCTCAGAAAGTGTTTCCTAATGGGGACTGGGAGAAGACTAGAGGCCGAAGACCATGAGGGGAGGGTGCAGTGGTTAAGAAAAAATGCCACCATCCCAAATGACTCAGGGGCAAGGAGAGCAGGTATAGTGGGTGCAAAGAGTGGCTAAGAGGCAGGGTGGGCGGCTCTTGGTTAGAGGGAAGTATGAGACAGGGCAGAGCAGAGGGGGACTTCCAGATTTCTGACCCAGACAGCTGAGCAGAGATAGGGGTCCTACACTGAGGTAAGGGGTAGAGGAGGAGAAGGGATGTCAGCTTTGAATATAGTGAGTTTGAAGTGGCTGGCTGAGTGACGATGTCTGGAGAGCGGAGAGTTAATGAGAAGGACGTCAGATGTAAGGAGACAGTGCTGGAGTTCCTGTTGTGGCTCAGCAGTAATGAATCCAACTAATATCCATGAGGATGCAGGTTCAATCCCTGGCCTCGCTCAGTGGGTTAAGGATCCAGTGATGCCATGAGCTG

g2P12

CCTCGGGCTCTCCCTATGTGAGTTCTTAAAGCCTTGGCCGCAAACTCGGACGCAGCCCAGGGAAGGTGGCAAACAGAACACTCTAGGATTTCCTGGTGGCTCATCTGTCATTTATCTCCAGAGCCCTTCGGTTGTCTGCCGTGCCGAACTTCATGTTCACATCCGCTCCTGACTTTCCGTTACTGAAACGAACCCAGAGCCGTCCACCATGGTCTGTCAACCCCAAATTTGGGAAAAGCCTCATGCATCAGGGGAAAAAAATAACAACAAAGGCATTTTGTTTTCCCAACTACAGTGCCTCCTGTTGATGGATTAGAATTCCATCATTAAATTATGTATGCTGACGTGGGAAAATCTGTTAAAGTGTATGTGGGAAAAAGCAGGATGCAAATTTATGTCAAACAAAGCAAGTGACAAATGAGTAAACAGCACAGAAAAATGTTTGGAAGGGAATACACTAAAATGCTGCCTTTGGGGGATGGGTAG

g2P13

TCCCAGGCTAGGAGTTGAATCAGAGCTGCAGCTGCCGGCCTAAGCCACCATCACAGCAACGCTGGATCTGAGGAGCATCTGTGACCTACACCGCAGCTTGAGGCAATGCCGGAGCCTTAACCCACTGAGAAAGGCCAGGGATCAAAACTGCATCCTCATGGACACTAGTCAGATTCTTAACCTGCTGAGCCACAATGGGAACTCCTAGAACAGTCCATCCTGATCTCCTAGAATAGGCACGTGGTCTAAGGGAGAATGGGGCAGCATAGAACATGGATCATGAACTCACACAAAACTAACTAGACTGGCTAACATGTACTAGATGTACTAGATGCAGGATTTCGTGGTCTATTGGGTGGACAATGACGATATCTGAGCAAGTACTGCAGTTGCCAGTCTCGGAGTCCCAACTTCTCATCAGATAAGTTTGCGGAGATTAAAACCTATAACCATCTTCAACAGTGACAAGCTGGCATTGATGA

**Data S2**

**TALEN pairs used in this study to target the pig *H11* locus**

TAL-H11-L1: TTCTTATGTTCCTGGAAG T

TAL-H11-L2: TCTTATGTTCCTGGAAGT T

TAL-H11-L3: CTTATGTTCCTGGAAGTT T

TAL-H11-R1: GTAGCCTATAAAACCCAG A

TAL-H11-R2: AGCCTATAAAACCCAGAG C

**Data S3**

**The sgRNA pair for CRISPR/Cas9n used in this study to target the pig *H11* locus (gray highlight is PAM sequence)**

CRISPR/Cas9n-L: AGATCAGGGTGGGCAGCTCTGGG

CRISPR/Cas9n-R: TTCCAGGAACATAAGAAAGTAGG

**Data S4**

**Vector sequences**

***pLHG-H11-GFP-DTA* sequence**

CTATAGTGAGTCGTATTACGCGCGCTCACTGGCCGTCGTTTTACAACGTCGTGACTGGGAAAACCCTGGCGTTACCCAACTTAATCGCCTTGCAGCACATCCCCCTTTCGCCAGCTGGCGTAATAGCGAAGAGGCCCGCACCGATCGCCCTTCCCAACAGTTGCGCAGCCTGAATGGCGAATGGGACGCGCCCTGTAGCGGCGCATTAAGCGCGGCGGGTGTGGTGGTTACGCGCAGCGTGACCGCTACACTTGCCAGCGCCCTAGCGCCCGCTCCTTTCGCTTTCTTCCCTTCCTTTCTCGCCACGTTCGCCGGCTTTCCCCGTCAAGCTCTAAATCGGGGGCTCCCTTTAGGGTTCCGATTTAGTGCTTTACGGCACCTCGACCCCAAAAAACTTGATTAGGGTGATGGTTCACGTAGTGGGCCATCGCCCTGATAGACGGTTTTTCGCCCTTTGACGTTGGAGTCCACGTTCTTTAATAGTGGACTCTTGTTCCAAACTGGAACAACACTCAACCCTATCTCGGTCTATTCTTTTGATTTATAAGGGATTTTGCCGATTTCGGCCTATTGGTTAAAAAATGAGCTGATTTAACAAAAATTTAACGCGAATTTTAACAAAATATTAACGCTTACAATTTAGGTGGCACTTTTCGGGGAAATGTGCGCGGAACCCCTATTTGTTTATTTTTCTAAATACATTCAAATATGTATCCGCTCATGAGACAATAACCCTGATAAATGCTTCAATAATATTGAAAAAGGAAGAGTATGAGTATTCAACATTTCCGTGTCGCCCTTATTCCCTTTTTTGCGGCATTTTGCCTTCCTGTTTTTGCTCACCCAGAAACGCTGGTGAAAGTAAAAGATGCTGAAGATCAGTTGGGTGCACGAGTGGGTTACATCGAACTGGATCTCAACAGCGGTAAGATCCTTGAGAGTTTTCGCCCCGAAGAACGTTTTCCAATGATGAGCACTTTTAAAGTTCTGCTATGTGGCGCGGTATTATCCCGTATTGACGCCGGGCAAGAGCAACTCGGTCGCCGCATACACTATTCTCAGAATGACTTGGTTGAGTACTCACCAGTCACAGAAAAGCATCTTACGGATGGCATGACAGTAAGAGAATTATGCAGTGCTGCCATAACCATGAGTGATAACACTGCGGCCAACTTACTTCTGACAACGATCGGAGGACCGAAGGAGCTAACCGCTTTTTTGCACAACATGGGGGATCATGTAACTCGCCTTGATCGTTGGGAACCGGAGCTGAATGAAGCCATACCAAACGACGAGCGTGACACCACGATGCCTGTAGCAATGGCAACAACGTTGCGCAAACTATTAACTGGCGAACTACTTACTCTAGCTTCCCGGCAACAATTAATAGACTGGATGGAGGCGGATAAAGTTGCAGGACCACTTCTGCGCTCGGCCCTTCCGGCTGGCTGGTTTATTGCTGATAAATCTGGAGCCGGTGAGCGTGGGTCTCGCGGTATCATTGCAGCACTGGGGCCAGATGGTAAGCCCTCCCGTATCGTAGTTATCTACACGACGGGGAGTCAGGCAACTATGGATGAACGAAATAGACAGATCGCTGAGATAGGTGCCTCACTGATTAAGCATTGGTAACTGTCAGACCAAGTTTACTCATATATACTTTAGATTGATTTAAAACTTCATTTTTAATTTAAAAGGATCTAGGTGAAGATCCTTTTTGATAATCTCATGACCAAAATCCCTTAACGTGAGTTTTCGTTCCACTGAGCGTCAGACCCCGTAGAAAAGATCAAAGGATCTTCTTGAGATCCTTTTTTTCTGCGCGTAATCTGCTGCTTGCAAACAAAAAAACCACCGCTACCAGCGGTGGTTTGTTTGCCGGATCAAGAGCTACCAACTCTTTTTCCGAAGGTAACTGGCTTCAGCAGAGCGCAGATACCAAATACTGTCCTTCTAGTGTAGCCGTAGTTAGGCCACCACTTCAAGAACTCTGTAGCACCGCCTACATACCTCGCTCTGCTAATCCTGTTACCAGTGGCTGCTGCCAGTGGCGATAAGTCGTGTCTTACCGGGTTGGACTCAAGACGATAGTTACCGGATAAGGCGCAGCGGTCGGGCTGAACGGGGGGTTCGTGCACACAGCCCAGCTTGGAGCGAACGACCTACACCGAACTGAGATACCTACAGCGTGAGCTATGAGAAAGCGCCACGCTTCCCGAAGGGAGAAAGGCGGACAGGTATCCGGTAAGCGGCAGGGTCGGAACAGGAGAGCGCACGAGGGAGCTTCCAGGGGGAAACGCCTGGTATCTTTATAGTCCTGTCGGGTTTCGCCACCTCTGACTTGAGCGTCGATTTTTGTGATGCTCGTCAGGGGGGCGGAGCCTATGGAAAAACGCCAGCAACGCGGCCTTTTTACGGTTCCTGGCCTTTTGCTGGCCTTTTGCTCACATGTTCTTTCCTGCGTTATCCCCTGATTCTGTGGATAACCGTATTACCGCCTTTGAGTGAGCTGATACCGCTCGCCGCAGCCGAACGACCGAGCGCAGCGAGTCAGTGAGCGAGGAAGCGGAAGAGCGCCCAATACGCAAACCGCCTCTCCCCGCGCGTTGGCCGATTCATTAATGCAGCTGGCACGACAGGTTTCCCGACTGGAAAGCGGGCAGTGAGCGCAACGCAATTAATGTGAGTTAGCTCACTCATTAGGCACCCCAGGCTTTACACTTTATGCTTCCGGCTCGTATGTTGTGTGGAATTGTGAGCGGATAACAATTTCACACAGGAAACAGCTATGACCATGATTACGCCAAGCTCGAAATTAACCCTCACTAAAGGGAACAAAAGCTGGAGCTACTTAAGGGCGCGCCCATTGAGCCACGAACAGAACTCCCTCTTACCAACTTATTACTACTAACTTCCCAAGTACTGGCTGCTCAGCTGCTTCCTTGGGCATGGGGGAGGGAGCACTATTTTTTCCTCTCCTGACTTCATCCTCTTCCTTTTAATTTCCATAAGGTTCCCTGTGGCCCTGTGCTTTTTTATTTTGAGGCCTTGCACATCCTTCTGGCCCTGATTGCTTCTCAACTCATCTTGTGCCTGCTGGACTTCCACCGTTGTTTCATGTATCTCGTTAGCTGAGATAGCACTTCCTCCTGCCCTTACCCTTTATCTGGCTCTTAGCTCCTGAAAACTGCATTATTAGCTTCCTCTTTTGCCTCTACTCTTACTCAACCAAAATTGTTTTAAGATCTGTGGATCTAGCTTCTGCTGTGCTATTCTTAGGAACACTTTTATTTCCTCTTAGCTCCATCTCACCAGTTATTGGCTAATGGCTTTGCTTGGTACCTACATCTGTACATTTCTTTCGTACTAGCTTCTAGACTGAAAAAGGACTGTTGGTTCAACATGAAAGGGAAGGAGGTAAAAGAGGACACACAGGAAAGATGGATTGGGATTCAGGTCTCTGCTGTTGTTACTTGAGATTGCTTTCTAGATTCTACTTGTGGAAACAAAAAGCCTTTGCGAGAATTCTAAACTGGAGTATTTCTGTAATTGAGGAGTCTTGCTCAGCAAATCCCACTTAGGGGACTAATGAAGTACCAGGAAGAGACAGACCATGCTCAATCCACAAAGCCAGGTTTTACTGAAATGTGACCTACTTTCTTATGCGATCGCCTGCCGAAAGAGTAATGTTGGCCGAGATAGGAGAAGACGATGATATCACGCTACGACGGAAACAGTACTATGGCCTCCTCCGAGGACGTCATCAAGGAGTTCATGCGCTTCAAGGTGCGCATGGAGGGCTCCGTGAACGGCCACGAGTTCGAGATCGAGGGCGAGGGCGAGGGCCGCCCCTACGAGGGCACCCAGACCGCCAAGCTGAAGGTGACCAAGGGCGGCCCCCTGCCCTTCGCCTGGGACATCCTGTCCCCTCAGTTCCAGTACGGCTCCAAGGCCTACGTGAAGCACCCCGCCGACATCCCCGACTACTTGAAGCTGTCCTTCCCCGAGGGCTTCAAGTGGGAGCGCGTGATGAACTTCGAGGACGGCGGCGTGGTGACCGTGACCCAGGACTCCTCCCTGCAGGACGGCGAGTTCATCTACAAGGTGAAGCTGCGCGGCACCAACTTCCCCTCCGACGGCCCCGTAATGCAGAAGAAGACCATGGGCTGGGAGGCCTCCACCGAGCGGATGTACCCCGAGGACGGCGCCCTGAAGGGCGAGATCAAGATGAGGCTGAAGCTGAAGGACGGCGGCCACTACGACGCCGAGGTCAAGACCACCTACATGGCCAAGAAGCCCGTGCAGCTGCCCGGCGCCTACAAGACCGACATCAAGCTGGACATCACCTCCCACAACGAGGACTACACCATCGTGGAACAGTACGAGCGCGCCGAGGGCCGCCACTCCACCGGCGCCTAAGAATGCAATTGTTGTTGTTAACTTGTTTATTGCAGCTTATAATGGTTACAAATAAAGCAATAGCATCACAAATTTCACAAATAAAGCATTTTTTTCACTGCATTCTAGTTGTGGTTTGTCCAAACTCATCAATGTATCTTATTAATTAAACGCGGTGGCGGCCGCATTACCCTGTTATCCCTAGAATTCGATGCTGAAGTTCCTATAGTTTCTAGAGTATAGGAACTTCGGTCATAACTTCGTATAGCATACATTATACGAAGTTATTCCGGATAAGATACATTGATGAGTTTGGACAAACCACAACTAGAATGCAGTGAAAAAAATGCTTTATTTGTGAAATTTGTGATGCTATTGCTTTATTTGTAACCATTATAAGCTGCAATAAACAAGTTGGGGTGGGCGAAGAACTCCAGCATGAGATCCCCGCGCTGGAGGATCATCCAGCCGGCGTCCCGGAAAACGATTCCGAAGCCCAACCTTTCATAGAAGGCGGCGGTGGAATCGAAATCTCGTGATGGCAGGTTGGGCGTCGCTTGGTCGGTCATTTCGAACCCCAGAGTCCCGCTCAGAAGAACTCGTCAAGAAGGCGATAGAAGGCGATGCGCTGCGAATCGGGAGCGGCGATACCGTAAAGCACGAGGAAGCGGTCAGCCCATTCGCCGCCAAGCTCTTCAGCAATATCACGGGTAGCCAACGCTATGTCCTGATAGCGGTCCGCCACACCCAGCCGGCCACAGTCGATGAATCCAGAAAAGCGGCCATTTTCCACCATGATATTCGGCAAGCAGGCATCGCCATGGGTCACGACGAGATCCTCGCCGTCGGGCATGCGCGCCTTGAGCCTGGCGAACAGTTCGGCTGGCGCGAGCCCCTGATGCTCTTCGTCCAGATCATCCTGATCGACAAGACCGGCTTCCATCCGAGTACGTGCTCGCTCGATGCGATGTTTCGCTTGGTGGTCGAATGGGCAGGTAGCCGGATCAAGCGTATGCAGCCGCCGCATTGCATCAGCCATGATGGATACTTTCTCGGCAGGAGCAAGGTGAGATGACAGGAGATCCTGCCCCGGCACTTCGCCCAATAGCAGCCAGTCCCTTCCCGCTTCAGTGACAACGTCGAGCACAGCTGCGCAAGGAACGCCCGTCGTGGCCAGCCACGATAGCCGCGCTGCCTCGTCCTGCAGTTCATTCAGGGCACCGGACAGGTCGGTCTTGACAAAAAGAACCGGGCGCCCCTGCGCTGACAGCCGGAACACGGCGGCATCAGAGCAGCCGATTGTCTGTTGTGCCCAGTCATAGCCGAATAGCCTCTCCACCCAAGCGGCCGGAGAACCTGCGTGCAATCCATCTTGTTCAATCATGCGAAACGATCCTCATGCTAGCTTATCATCGTGTTTTTCAAAGGAAAACCACGTCCCCGTGGTTCGGGGGGCCTAGACGTTTTTTTAACCTCGACTAAACACATGTAAAGCATGTGCACCGAGGCCCCAGATCAGATCCCATACAATGGGGTACCTTCTGGGCATCCTTCAGCCCCTTGTTGAATACGCTTGAGGAGAGCCATTTGACTCTTTCCACAACTATCCAACTCACAACGTGGCACTGGGGTTGTGCCGCCTTTGCAGGTGTATCTTATACACGTGGCTTTTGGCCGCAGAGGCACCTGTCGCCAGGTGGGGGGTTCCGCTGCCTGCAAAGGGTCGCTACAGACGTTGTTTGTCTTCAAGAAGCTTCCAGAGGAACTGCTTCCTTCACGACATTCAACAGACCTTGCATTCCTTTGGCGAGAGGGGAAAGACCCCTAGGAATGCTCGTCAAGAAGACAGGGCCAGGTTTCCGGGCCCTCACATTGCCAAAAGACGGCAATATGGTGGAAAATAACATATAGACAAACGCACACCGGCCTTATTCCAAGCGGCTTCGGCCAGTAACGTTAGGGGGGGGGGGGGAGAGGGGCGGAATTGGATCCGATATCTTACTTGTACAGCTCGTCCATGCCGAGAGTGATCCCGGCGGCGGTCACGAACTCCAGCAGGACCATGTGATCGCGCTTCTCGTTGGGGTCTTTGCTCAGGGCGGACTGGGTGCTCAGGTAGTGGTTGTCGGGCAGCAGCACGGGGCCGTCGCCGATGGGGGTGTTCTGCTGGTAGTGGTCGGCGAGCTGCACGCTGCCGTCCTCGATGTTGTGGCGGATCTTGAAGTTCACCTTGATGCCGTTCTTCTGCTTGTCGGCCATGATATAGACGTTGTGGCTGTTGTAGTTGTACTCCAGCTTGTGCCCCAGGATGTTGCCGTCCTCCTTGAAGTCGATGCCCTTCAGCTCGATGCGGTTCACCAGGGTGTCGCCCTCGAACTTCACCTCGGCGCGGGTCTTGTAGTTGCCGTCGTCCTTGAAGAAGATGGTGCGCTCCTGGACGTAGCCTTCGGGCATGGCGGACTTGAAGAAGTCGTGCTGCTTCATGTGGTCGGGGTAGCGGCTGAAGCACTGCACGCCGTAGGTCAGGGTGGTCACGAGGGTGGGCCAGGGCACGGGCAGCTTGCCGGTGGTGCAGATGAACTTCAGGGTCAGCTTGCCGTAGGTGGCATCGCCCTCGCCCTCGCCGGACACGCTGAACTTGTGGCCGTTTACGTCGCCGTCCAGCTCGACCAGGATGGGCACCACCCCGGTGAACAGCTCCTCGCCCTTGCTCACCATCTTAAGGATCTGACGGTTCACTAAACCAGCTCTGCTTATATAGACCTCCCACCGTACACGCCTACCGCCCATTTGCGTCAATGGGGCGGAGTTGTTACGACATTTTGGAAAGTCCCGTTGATTTTGGTGCCAAAACAAACTCCCATTGACGTCAATGGGGTGGAGACTTGGAAATCCCCGTGAGTCAAACCGCTATCCACGCCCATTGATGTACTGCCAAAACCGCATCACCATGGTAATAGCGATGACTAATACGTAGATGTACTGCCAAGTAGGAAAGTCCCATAAGGTCATGTACTGGGCATAATGCCAGGCGGGCCATTTACCGTCATTGACGTCAATAGGGGGCGTACTTGGCATATGATACACTTGATGTACTGCCAAGTGGGCAGTTTACCGTAAATACTCCACCCATTGACGTCAATGGAAAGTCCCTATTGGCGTTACTATGGGAACATACGTCATTATTGACGTCAATGGGCGGGGGTCGTTGGGCGGTCAGCCAGGCGGGCCATTTACCGTAAGTTATGTAACGCGGAACTCCATATATGGGCTATGAACTAATGACCCCGTAATTGAGATCTGAAGTTCCTATAGTTTCTAGAGTATAGGAACTTCGGTCATAACTTCGTATAGCATACATTATACGAAGTTATACGCGTTTCCCGAGGCTGAGTTAGTTGGTCCAGCCAGTGATTGAGTTGCGTGCGGAGGGCTTCTTATCTTAGTTTTATAGGCTACACTGTTAACACTCAGGCTGTTTTCTACCGTTTAGTCAAAATATAGTCACCTTGCCTGCTTCACCTGTCCATCAGAGAATGGCCTCATTAATTGACTCTCTAGTATGAAGTCAAAGTAGCTTTGGTGGCCCTAAATGGACAAGTATCAAGAGACTGGGTGAATTGAGGAGCTTGAGACTGTCACCTCAGATCGAAAAGACTGAAAAATCACCTCAGATCAAAAAGACTGAAAAATCTTCAGTCTGGAAAGGGGACTCAAAACCATAATTAGAGTATTCTGGTAGAATCCTTTTCTCCACTGTTATTCATACAGTTAAGGTGAATAACTAAAAGTAATTGTGAGCTGAGGAGTAAGATACAACACACAAGGAATCAGTTAACAGAGTCTCGAGTGAAATTATAAATGGAAAGAATTATGACTTGAATCATAACTCTGAGGCCCCATTTTCCCTAACAACTTTTGTCCCAATAAACGTGGGTATTTGTTTGGGAGAAACTATCATATACATGATTACCCAGTAAACAGACTGTTTACTAAGTGGGTTTAATTTTAGAAATTGCGCGCTGCAATCTGGTATTAACCATACAACTACCTACCTATAGGGTCAGCCCAGCCTGAACTATCCCATTGGGGTCTTTATTAAGGCTCAAGAAACGGCCATAGCTTCTTCCTTTAAAATGAGTGTTTATTTCTATGAGCTTTAAAGAAAAAAACAGATAATTTCCCTCAACCTACTGAAGAGGAAGGGATTCAGGAAGAAATAAACACAACAATGCCATTCACTTCAGGCCGGCCTCTAGAATGCATGTTTAAACAGGCCGCGGGAATTCGATTATCGAATTCTACCGGGTAGGGGAGGCGCTTTTCCCAAGGCAGTCTGGAGCATGCGCTTTAGCAGCCCCGCTGGGCACTTGGCGCTACACAAGTGGCCTCTGGCCTCGCACACATTCCACATCCACCGGTAGGCGCCAACCGGCTCCGTTCTTTGGTGGCCCCTTCGCGCCACCTTCTACTCCTCCCCTAGTCAGGAAGTTCCCCCCCGCCCCGCAGCTCGCGTCGTGCAGGACGTGACAAATGGAAGTAGCACGTCTCACTAGTCTCGTGCAGATGGACAGCACCGCTGAGCAATGGAAGCGGGTAGGCCTTTGGGGCAGCGGCCAATAGCAGCTTTGCTCCTTCGCTTTCTGGGCTCAGAGGCTGGGAAGGGGTGGGTCCGGGGGCGGGCTCAGGGGCGGGCTCAGGGGCGGGGCGGGCGCCCGAAGGTCCTCCGGAGGCCCGGCATTCTGCACGCTTCAAAAGCGCACGTCTGCCGCGCTGTTCTCCTCTTCCTCATCTCCGGGCCTTTCGACCTGCAGGTCCTCGCCATGGATCCTGATGATGTTGTTGATTCTTCTAAATCTTTTGTGATGGAAAACTTTTCTTCGTACCACGGGACTAAACCTGGTTATGTAGATTCCATTCAAAAAGGTATACAAAAGCCAAAATCTGGTACACAAGGAAATTATGACGATGATTGGAAAGGGTTTTATAGTACCGACAATAAATACGACGCTGCGGGATACTCTGTAGATAATGAAAACCCGCTCTCTGGAAAAGCTGGAGGCGTGGTCAAAGTGACGTATCCAGGACTGACGAAGGTTCTCGCACTAAAAGTGGATAATGCCGAAACTATTAAGAAAGAGTTAGGTTTAAGTCTCACTGAACCGTTGATGGAGCAAGTCGGAACGGAAGAGTTTATCAAAAGGTTCGGTGATGGTGCTTCGCGTGTAGTGCTCAGCCTTCCCTTCGCTGAGGGGAGTTCTAGCGTTGAATATATTAATAACTGGGAACAGGCGAAAGCGTTAAGCGTAGAACTTGAGATTAATTTTGAAACCCGTGGAAAACGTGGCCAAGATGCGATGTATGAGTATATGGCTCAAGCCTGTGCAGGAAATCGTGTCAGGCGATCTCTTTGTGAAGGAACCTTACTTCTGTGGTGTGACATAATTGGACAAACTACCTACAGAGATTTAAAGCTCTAAGGTAAATATAAAATTTTTAAGTGTATAATGTGTTAAACTACTGATTCTAATTGTTTGTGTATTTTAGATTCCAACCTATGGAACTGATGAATGGGAGCAGTGGTGGAATGCAGATCCTAGAGCTCGCTGATCAGCCTCGACTGTGCCTTCTAGTTGCCAGCCATCTATTGTTTGCCCCTCCCCCGTGCCTTCCTTGACCCTGGAAGGTGCCACTCCCACTGTCCTTTCCTAATAAAATGAGGAAATTGCATCGCATTGTCTGAGTAGGTGTCATTCTATTCTGGGGGGTGGGGTGGGGCAGGACAGCAAGGGGGAGGATTGGGAAGACAATAGCAGGCATGCTGGGGATGCGGTGGGCTCTATGGCTTCTGAGGCGGAAAGAACCAGCTGGGGCTCGAGGGGGGGCCCGGTACCCAATTCGCC

***puc-H11-GFP* sequence**

TCGCGCGTTTCGGTGATGACGGTGAAAACCTCTGACACATGCAGCTCCCGGAGACGGTCACAGCTTGTCTGTAAGCGGATGCCGGGAGCAGACAAGCCCGTCAGGGCGCGTCAGCGGGTGTTGGCGGGTGTCGGGGCTGGCTTAACTATGCGGCATCAGAGCAGATTGTACTGAGAGTGCACCATATGCGGTGTGAAATACCGCACAGATGCGTAAGGAGAAAATACCGCATCAGGCGCCATTCGCCATTCAGGCTGCGCAACTGTTGGGAAGGGCGATCGGTGCGGGCCTCTTCGCTATTACGCCAGCTGGCGAAAGGGGGATGTGCTGCAAGGCGATTAAGTTGGGTAACGCCAGGGTTTTCCCAGTCACGACGTTGTAAAACGACGGCCAGTGAATTGCGGCCTTTGGCGCGCCCATTGAGCCACGAACAGAACTCCCTCTTACCAACTTATTACTACTAACTTCCCAAGTACTGGCTGCTCAGCTGCTTCCTTGGGCATGGGGGAGGGAGCACTATTTTTTCCTCTCCTGACTTCATCCTCTTCCTTTTAATTTCCATAAGGTTCCCTGTGGCCCTGTGCTTTTTTATTTTGAGGCCTTGCACATCCTTCTGGCCCTGATTGCTTCTCAACTCATCTTGTGCCTGCTGGACTTCCACCGTTGTTTCATGTATCTCGTTAGCTGAGATAGCACTTCCTCCTGCCCTTACCCTTTATCTGGCTCTTAGCTCCTGAAAACTGCATTATTAGCTTCCTCTTTTGCCTCTACTCTTACTCAACCAAAATTGTTTTAAGATCTGTGGATCTAGCTTCTGCTGTGCTATTCTTAGGAACACTTTTATTTCCTCTTAGCTCCATCTCACCAGTTATTGGCTAATGGCTTTGCTTGGTACCTACATCTGTACATTTCTTTCGTACTAGCTTCTAGACTGAAAAAGGACTGTTGGTTCAACATGAAAGGGAAGGAGGTAAAAGAGGACACACAGGAAAGATGGATTGGGATTCAGGTCTCTGCTGTTGTTACTTGAGATTGCTTTCTAGATTCTACTTGTGGAAACAAAAAGCCTTTGCGAGAATTCTAAACTGGAGTATTTCTGTAATTGAGGAGTCTTGCTCAGCAAATCCCACTTAGGGGACTAATGAAGTACCAGGAAGAGACAGACCATGCTCAATCCACAAAGCCAGGTTTTACTGAAATGTGACCTACTTTCTTATGCGATCGCCTGCCGAAAGAGTAATGTTGGCCGAGATAGGAGAAGACGATGATATCACGCTACGACGGAAACAGTACTATGGCCTCCTCCGAGGACGTCATCAAGGAGTTCATGCGCTTCAAGGTGCGCATGGAGGGCTCCGTGAACGGCCACGAGTTCGAGATCGAGGGCGAGGGCGAGGGCCGCCCCTACGAGGGCACCCAGACCGCCAAGCTGAAGGTGACCAAGGGCGGCCCCCTGCCCTTCGCCTGGGACATCCTGTCCCCTCAGTTCCAGTACGGCTCCAAGGCCTACGTGAAGCACCCCGCCGACATCCCCGACTACTTGAAGCTGTCCTTCCCCGAGGGCTTCAAGTGGGAGCGCGTGATGAACTTCGAGGACGGCGGCGTGGTGACCGTGACCCAGGACTCCTCCCTGCAGGACGGCGAGTTCATCTACAAGGTGAAGCTGCGCGGCACCAACTTCCCCTCCGACGGCCCCGTAATGCAGAAGAAGACCATGGGCTGGGAGGCCTCCACCGAGCGGATGTACCCCGAGGACGGCGCCCTGAAGGGCGAGATCAAGATGAGGCTGAAGCTGAAGGACGGCGGCCACTACGACGCCGAGGTCAAGACCACCTACATGGCCAAGAAGCCCGTGCAGCTGCCCGGCGCCTACAAGACCGACATCAAGCTGGACATCACCTCCCACAACGAGGACTACACCATCGTGGAACAGTACGAGCGCGCCGAGGGCCGCCACTCCACCGGCGCCTAAGAATGCAATTGTTGTTGTTAACTTGTTTATTGCAGCTTATAATGGTTACAAATAAAGCAATAGCATCACAAATTTCACAAATAAAGCATTTTTTTCACTGCATTCTAGTTGTGGTTTGTCCAAACTCATCAATGTATCTTATTAATTAAACGCGGTGGCGGCCGCATTACCCTGTTATCCCTAGAATTCGATGCTGAAGTTCCTATAGTTTCTAGAGTATAGGAACTTCGGTCATAACTTCGTATAGCATACATTATACGAAGTTATTCCGGATAAGATACATTGATGAGTTTGGACAAACCACAACTAGAATGCAGTGAAAAAAATGCTTTATTTGTGAAATTTGTGATGCTATTGCTTTATTTGTAACCATTATAAGCTGCAATAAACAAGTTGGGGTGGGCGAAGAACTCCAGCATGAGATCCCCGCGCTGGAGGATCATCCAGCCGGCGTCCCGGAAAACGATTCCGAAGCCCAACCTTTCATAGAAGGCGGCGGTGGAATCGAAATCTCGTGATGGCAGGTTGGGCGTCGCTTGGTCGGTCATTTCGAACCCCAGAGTCCCGCTCAGAAGAACTCGTCAAGAAGGCGATAGAAGGCGATGCGCTGCGAATCGGGAGCGGCGATACCGTAAAGCACGAGGAAGCGGTCAGCCCATTCGCCGCCAAGCTCTTCAGCAATATCACGGGTAGCCAACGCTATGTCCTGATAGCGGTCCGCCACACCCAGCCGGCCACAGTCGATGAATCCAGAAAAGCGGCCATTTTCCACCATGATATTCGGCAAGCAGGCATCGCCATGGGTCACGACGAGATCCTCGCCGTCGGGCATGCGCGCCTTGAGCCTGGCGAACAGTTCGGCTGGCGCGAGCCCCTGATGCTCTTCGTCCAGATCATCCTGATCGACAAGACCGGCTTCCATCCGAGTACGTGCTCGCTCGATGCGATGTTTCGCTTGGTGGTCGAATGGGCAGGTAGCCGGATCAAGCGTATGCAGCCGCCGCATTGCATCAGCCATGATGGATACTTTCTCGGCAGGAGCAAGGTGAGATGACAGGAGATCCTGCCCCGGCACTTCGCCCAATAGCAGCCAGTCCCTTCCCGCTTCAGTGACAACGTCGAGCACAGCTGCGCAAGGAACGCCCGTCGTGGCCAGCCACGATAGCCGCGCTGCCTCGTCCTGCAGTTCATTCAGGGCACCGGACAGGTCGGTCTTGACAAAAAGAACCGGGCGCCCCTGCGCTGACAGCCGGAACACGGCGGCATCAGAGCAGCCGATTGTCTGTTGTGCCCAGTCATAGCCGAATAGCCTCTCCACCCAAGCGGCCGGAGAACCTGCGTGCAATCCATCTTGTTCAATCATGCGAAACGATCCTCATGCTAGCTTATCATCGTGTTTTTCAAAGGAAAACCACGTCCCCGTGGTTCGGGGGGCCTAGACGTTTTTTTAACCTCGACTAAACACATGTAAAGCATGTGCACCGAGGCCCCAGATCAGATCCCATACAATGGGGTACCTTCTGGGCATCCTTCAGCCCCTTGTTGAATACGCTTGAGGAGAGCCATTTGACTCTTTCCACAACTATCCAACTCACAACGTGGCACTGGGGTTGTGCCGCCTTTGCAGGTGTATCTTATACACGTGGCTTTTGGCCGCAGAGGCACCTGTCGCCAGGTGGGGGGTTCCGCTGCCTGCAAAGGGTCGCTACAGACGTTGTTTGTCTTCAAGAAGCTTCCAGAGGAACTGCTTCCTTCACGACATTCAACAGACCTTGCATTCCTTTGGCGAGAGGGGAAAGACCCCTAGGAATGCTCGTCAAGAAGACAGGGCCAGGTTTCCGGGCCCTCACATTGCCAAAAGACGGCAATATGGTGGAAAATAACATATAGACAAACGCACACCGGCCTTATTCCAAGCGGCTTCGGCCAGTAACGTTAGGGGGGGGGGGGGAGAGGGGCGGAATTGGATCCGATATCTTACTTGTACAGCTCGTCCATGCCGAGAGTGATCCCGGCGGCGGTCACGAACTCCAGCAGGACCATGTGATCGCGCTTCTCGTTGGGGTCTTTGCTCAGGGCGGACTGGGTGCTCAGGTAGTGGTTGTCGGGCAGCAGCACGGGGCCGTCGCCGATGGGGGTGTTCTGCTGGTAGTGGTCGGCGAGCTGCACGCTGCCGTCCTCGATGTTGTGGCGGATCTTGAAGTTCACCTTGATGCCGTTCTTCTGCTTGTCGGCCATGATATAGACGTTGTGGCTGTTGTAGTTGTACTCCAGCTTGTGCCCCAGGATGTTGCCGTCCTCCTTGAAGTCGATGCCCTTCAGCTCGATGCGGTTCACCAGGGTGTCGCCCTCGAACTTCACCTCGGCGCGGGTCTTGTAGTTGCCGTCGTCCTTGAAGAAGATGGTGCGCTCCTGGACGTAGCCTTCGGGCATGGCGGACTTGAAGAAGTCGTGCTGCTTCATGTGGTCGGGGTAGCGGCTGAAGCACTGCACGCCGTAGGTCAGGGTGGTCACGAGGGTGGGCCAGGGCACGGGCAGCTTGCCGGTGGTGCAGATGAACTTCAGGGTCAGCTTGCCGTAGGTGGCATCGCCCTCGCCCTCGCCGGACACGCTGAACTTGTGGCCGTTTACGTCGCCGTCCAGCTCGACCAGGATGGGCACCACCCCGGTGAACAGCTCCTCGCCCTTGCTCACCATCTTAAGGATCTGACGGTTCACTAAACCAGCTCTGCTTATATAGACCTCCCACCGTACACGCCTACCGCCCATTTGCGTCAATGGGGCGGAGTTGTTACGACATTTTGGAAAGTCCCGTTGATTTTGGTGCCAAAACAAACTCCCATTGACGTCAATGGGGTGGAGACTTGGAAATCCCCGTGAGTCAAACCGCTATCCACGCCCATTGATGTACTGCCAAAACCGCATCACCATGGTAATAGCGATGACTAATACGTAGATGTACTGCCAAGTAGGAAAGTCCCATAAGGTCATGTACTGGGCATAATGCCAGGCGGGCCATTTACCGTCATTGACGTCAATAGGGGGCGTACTTGGCATATGATACACTTGATGTACTGCCAAGTGGGCAGTTTACCGTAAATACTCCACCCATTGACGTCAATGGAAAGTCCCTATTGGCGTTACTATGGGAACATACGTCATTATTGACGTCAATGGGCGGGGGTCGTTGGGCGGTCAGCCAGGCGGGCCATTTACCGTAAGTTATGTAACGCGGAACTCCATATATGGGCTATGAACTAATGACCCCGTAATTGAGATCTGAAGTTCCTATAGTTTCTAGAGTATAGGAACTTCGGTCATAACTTCGTATAGCATACATTATACGAAGTTATACGCGTTTCCCGAGGCTGAGTTAGTTGGTCCAGCCAGTGATTGAGTTGCGTGCGGAGGGCTTCTTATCTTAGTTTTATAGGCTACACTGTTAACACTCAGGCTGTTTTCTACCGTTTAGTCAAAATATAGTCACCTTGCCTGCTTCACCTGTCCATCAGAGAATGGCCTCATTAATTGACTCTCTAGTATGAAGTCAAAGTAGCTTTGGTGGCCCTAAATGGACAAGTATCAAGAGACTGGGTGAATTGAGGAGCTTGAGACTGTCACCTCAGATCGAAAAGACTGAAAAATCACCTCAGATCAAAAAGACTGAAAAATCTTCAGTCTGGAAAGGGGACTCAAAACCATAATTAGAGTATTCTGGTAGAATCCTTTTCTCCACTGTTATTCATACAGTTAAGGTGAATAACTAAAAGTAATTGTGAGCTGAGGAGTAAGATACAACACACAAGGAATCAGTTAACAGAGTCTCGAGTGAAATTATAAATGGAAAGAATTATGACTTGAATCATAACTCTGAGGCCCCATTTTCCCTAACAACTTTTGTCCCAATAAACGTGGGTATTTGTTTGGGAGAAACTATCATATACATGATTACCCAGTAAACAGACTGTTTACTAAGTGGGTTTAATTTTAGAAATTGCGCGCTGCAATCTGGTATTAACCATACAACTACCTACCTATAGGGTCAGCCCAGCCTGAACTATCCCATTGGGGTCTTTATTAAGGCTCAAGAAACGGCCATAGCTTCTTCCTTTAAAATGAGTGTTTATTTCTATGAGCTTTAAAGAAAAAAACAGATAATTTCCCTCAACCTACTGAAGAGGAAGGGATTCAGGAAGAAATAAACACAACAATGCCATTCACTTCAGGCCGGCCATGCATAGCTTGGCGTAATCATGGTCATAGCTGTTTCCTGTGTGAAATTGTTATCCGCTCACAATTCCACACAACATACGAGCCGGAAGCATAAAGTGTAAAGCCTGGGGTGCCTAATGAGTGAGCTAACTCACATTAATTGCGTTGCGCTCACTGCCCGCTTTCCAGTCGGGAAACCTGTCGTGCCAGCTGCATTAATGAATCGGCCAACGCGCGGGGAGAGGCGGTTTGCGTATTGGGCGCTCTTCCGCTTCCTCGCTCACTGACTCGCTGCGCTCGGTCGTTCGGCTGCGGCGAGCGGTATCAGCTCACTCAAAGGCGGTAATACGGTTATCCACAGAATCAGGGGATAACGCAGGAAAGAACATGTGAGCAAAAGGCCAGCAAAAGGCCAGGAACCGTAAAAAGGCCGCGTTGCTGGCGTTTTTCCATAGGCTCCGCCCCCCTGACGAGCATCACAAAAATCGACGCTCAAGTCAGAGGTGGCGAAACCCGACAGGACTATAAAGATACCAGGCGTTTCCCCCTGGAAGCTCCCTCGTGCGCTCTCCTGTTCCGACCCTGCCGCTTACCGGATACCTGTCCGCCTTTCTCCCTTCGGGAAGCGTGGCGCTTTCTCAATGCTCACGCTGTAGGTATCTCAGTTCGGTGTAGGTCGTTCGCTCCAAGCTGGGCTGTGTGCACGAACCCCCCGTTCAGCCCGACCGCTGCGCCTTATCCGGTAACTATCGTCTTGAGTCCAACCCGGTAAGACACGACTTATCGCCACTGGCAGCAGCCACTGGTAACAGGATTAGCAGAGCGAGGTATGTAGGCGGTGCTACAGAGTTCTTGAAGTGGTGGCCTAACTACGGCTACACTAGAAGGACAGTATTTGGTATCTGCGCTCTGCTGAAGCCAGTTACCTTCGGAAAAAGAGTTGGTAGCTCTTGATCCGGCAAACAAACCACCGCTGGTAGCGGTGGTTTTTTTGTTTGCAAGCAGCAGATTACGCGCAGAAAAAAAGGATCTCAAGAAGATCCTTTGATCTTTTCTACGGGGTCTGACGCTCAGTGGAACGAAAACTCACGTTAAGGGATTTTGGTCATGAGATTATCAAAAAGGATCTTCACCTAGATCCTTTTAAATTAAAAATGAAGTTTTAAATCAATCTAAAGTATATATGAGTAAACTTGGTCTGACAGTTACCAATGCTTAATCAGTGAGGCACCTATCTCAGCGATCTGTCTATTTCGTTCATCCATAGTTGCCTGACTCCCCGTCGTGTAGATAACTACGATACGGGAGGGCTTACCATCTGGCCCCAGTGCTGCAATGATACCGCGAGACCCACGCTCACCGGCTCCAGATTTATCAGCAATAAACCAGCCAGCCGGAAGGGCCGAGCGCAGAAGTGGTCCTGCAACTTTATCCGCCTCCATCCAGTCTATTAATTGTTGCCGGGAAGCTAGAGTAAGTAGTTCGCCAGTTAATAGTTTGCGCAACGTTGTTGCCATTGCTACAGGCATCGTGGTGTCACGCTCGTCGTTTGGTATGGCTTCATTCAGCTCCGGTTCCCAACGATCAAGGCGAGTTACATGATCCCCCATGTTGTGCAAAAAAGCGGTTAGCTCCTTCGGTCCTCCGATCGTTGTCAGAAGTAAGTTGGCCGCAGTGTTATCACTCATGGTTATGGCAGCACTGCATAATTCTCTTACTGTCATGCCATCCGTAAGATGCTTTTCTGTGACTGGTGAGTACTCAACCAAGTCATTCTGAGAATAGTGTATGCGGCGACCGAGTTGCTCTTGCCCGGCGTCAATACGGGATAATACCGCGCCACATAGCAGAACTTTAAAAGTGCTCATCATTGGAAAACGTTCTTCGGGGCGAAAACTCTCAAGGATCTTACCGCTGTTGAGATCCAGTTCGATGTAACCCACTCGTGCACCCAACTGATCTTCAGCATCTTTTACTTTCACCAGCGTTTCTGGGTGAGCAAAAACAGGAAGGCAAAATGCCGCAAAAAAGGGAATAAGGGCGACACGGAAATGTTGAATACTCATACTCTTCCTTTTTCAATATTATTGAAGCATTTATCAGGGTTATTGTCTCATGAGCGGATACATATTTGAATGTATTTAGAAAAATAAACAAATAGGGGTTCCGCGCACATTTCCCCGAAAAGTGCCACCTGACGTCTAAGAAACCATTATTATCATGACATTAACCTATAAAAATAGGCGTATCACGAGGCCCTTTCGTC

***pLHG-H11-long-DTA* sequence**

CTATAGTGAGTCGTATTACAATTCACTGGCCGTCGTTTTACAACGTCGTGACTGGGAAAACCCTGGCGTTACCCAACTTAATCGCCTTGCAGCACATCCCCCTTTCGCCAGCTGGCGTAATAGCGAAGAGGCCCGCACCGATCGCCCTTCCCAACAGTTGCGCAGCCTGAATGGCGAATGGGACGCGCCCTGTAGCGGCGCATTAAGCGCGGCGGGTGTGGTGGTTACGCGCAGCGTGACCGCTACACTTGCCAGCGCCCTAGCGCCCGCTCCTTTCGCTTTCTTCCCTTCCTTTCTCGCCACGTTCGCCGGCTTTCCCCGTCAAGCTCTAAATCGGGGGCTCCCTTTAGGGTTCCGATTTAGTGCTTTACGGCACCTCGACCCCAAAAAACTTGATTAGGGTGATGGTTCACGTAGTGGGCCATCGCCCTGATAGACGGTTTTTCGCCCTTTGACGTTGGAGTCCACGTTCTTTAATAGTGGACTCTTGTTCCAAACTGGAACAACACTCAACCCTATCTCGGTCTATTCTTTTGATTTATAAGGGATTTTGCCGATTTCGGCCTATTGGTTAAAAAATGAGCTGATTTAACAAAAATTTAACGCGAATTTTAACAAAATATTAACGCTTACAATTTAGGTGGCACTTTTCGGGGAAATGTGCGCGGAACCCCTATTTGTTTATTTTTCTAAATACATTCAAATATGTATCCGCTCATGAGACAATAACCCTGATAAATGCTTCAATAATATTGAAAAAGGAAGAGTATGAGTATTCAACATTTCCGTGTCGCCCTTATTCCCTTTTTTGCGGCATTTTGCCTTCCTGTTTTTGCTCACCCAGAAACGCTGGTGAAAGTAAAAGATGCTGAAGATCAGTTGGGTGCACGAGTGGGTTACATCGAACTGGATCTCAACAGCGGTAAGATCCTTGAGAGTTTTCGCCCCGAAGAACGTTTTCCAATGATGAGCACTTTTAAAGTTCTGCTATGTGGCGCGGTATTATCCCGTATTGACGCCGGGCAAGAGCAACTCGGTCGCCGCATACACTATTCTCAGAATGACTTGGTTGAGTACTCACCAGTCACAGAAAAGCATCTTACGGATGGCATGACAGTAAGAGAATTATGCAGTGCTGCCATAACCATGAGTGATAACACTGCGGCCAACTTACTTCTGACAACGATCGGAGGACCGAAGGAGCTAACCGCTTTTTTGCACAACATGGGGGATCATGTAACTCGCCTTGATCGTTGGGAACCGGAGCTGAATGAAGCCATACCAAACGACGAGCGTGACACCACGATGCCTGTAGCAATGGCAACAACGTTGCGCAAACTATTAACTGGCGAACTACTTACTCTAGCTTCCCGGCAACAATTAATAGACTGGATGGAGGCGGATAAAGTTGCAGGACCACTTCTGCGCTCGGCCCTTCCGGCTGGCTGGTTTATTGCTGATAAATCTGGAGCCGGTGAGCGTGGGTCTCGCGGTATCATTGCAGCACTGGGGCCAGATGGTAAGCCCTCCCGTATCGTAGTTATCTACACGACGGGGAGTCAGGCAACTATGGATGAACGAAATAGACAGATCGCTGAGATAGGTGCCTCACTGATTAAGCATTGGTAACTGTCAGACCAAGTTTACTCATATATACTTTAGATTGATTTAAAACTTCATTTTTAATTTAAAAGGATCTAGGTGAAGATCCTTTTTGATAATCTCATGACCAAAATCCCTTAACGTGAGTTTTCGTTCCACTGAGCGTCAGACCCCGTAGAAAAGATCAAAGGATCTTCTTGAGATCCTTTTTTTCTGCGCGTAATCTGCTGCTTGCAAACAAAAAAACCACCGCTACCAGCGGTGGTTTGTTTGCCGGATCAAGAGCTACCAACTCTTTTTCCGAAGGTAACTGGCTTCAGCAGAGCGCAGATACCAAATACTGTCCTTCTAGTGTAGCCGTAGTTAGGCCACCACTTCAAGAACTCTGTAGCACCGCCTACATACCTCGCTCTGCTAATCCTGTTACCAGTGGCTGCTGCCAGTGGCGATAAGTCGTGTCTTACCGGGTTGGACTCAAGACGATAGTTACCGGATAAGGCGCAGCGGTCGGGCTGAACGGGGGGTTCGTGCACACAGCCCAGCTTGGAGCGAACGACCTACACCGAACTGAGATACCTACAGCGTGAGCTATGAGAAAGCGCCACGCTTCCCGAAGGGAGAAAGGCGGACAGGTATCCGGTAAGCGGCAGGGTCGGAACAGGAGAGCGCACGAGGGAGCTTCCAGGGGGAAACGCCTGGTATCTTTATAGTCCTGTCGGGTTTCGCCACCTCTGACTTGAGCGTCGATTTTTGTGATGCTCGTCAGGGGGGCGGAGCCTATGGAAAAACGCCAGCAACGCGGCCTTTTTACGGTTCCTGGCCTTTTGCTGGCCTTTTGCTCACATGTTCTTTCCTGCGTTATCCCCTGATTCTGTGGATAACCGTATTACCGCCTTTGAGTGAGCTGATACCGCTCGCCGCAGCCGAACGACCGAGCGCAGCGAGTCAGTGAGCGAGGAAGCGGAAGAGCGCCCAATACGCAAACCGCCTCTCCCCGCGCGTTGGCCGATTCATTAATGCAGCTGGCACGACAGGTTTCCCGACTGGAAAGCGGGCAGTGAGCGCAACGCAATTAATGTGAGTTAGCTCACTCATTAGGCACCCCAGGCTTTACACTTTATGCTTCCGGCTCGTATGTTGTGTGGAATTGTGAGCGGATAACAATTTCACACAGGAAACAGCTATGACCATGATTACGCCAAGCTCGAAATTAACCCTCACTAAAGGGAACAAAAGCTGGAGCTACTTAAGGGCGCGCCCATTGAGCCACGAACAGAACTCCCTCTTACCAACTTATTACTACTAACTTCCCAAGTACTGGCTGCTCAGCTGCTTCCTTGGGCATGGGGGAGGGAGCACTATTTTTTCCTCTCCTGACTTCATCCTCTTCCTTTTAATTTCCATAAGGTTCCCTGTGGCCCTGTGCTTTTTTATTTTGAGGCCTTGCACATCCTTCTGGCCCTGATTGCTTCTCAACTCATCTTGTGCCTGCTGGACTTCCACCGTTGTTTCATGTATCTCGTTAGCTGAGATAGCACTTCCTCCTGCCCTTACCCTTTATCTGGCTCTTAGCTCCTGAAAACTGCATTATTAGCTTCCTCTTTTGCCTCTACTCTTACTCAACCAAAATTGTTTTAAGATCTGTGGATCTAGCTTCTGCTGTGCTATTCTTAGGAACACTTTTATTTCCTCTTAGCTCCATCTCACCAGTTATTGGCTAATGGCTTTGCTTGGTACCTACATCTGTACATTTCTTTCGTACTAGCTTCTAGACTGAAAAAGGACTGTTGGTTCAACATGAAAGGGAAGGAGGTAAAAGAGGACACACAGGAAAGATGGATTGGGATTCAGGTCTCTGCTGTTGTTACTTGAGATTGCTTTCTAGATTCTACTTGTGGAAACAAAAAGCCTTTGCGAGAATTCTAAACTGGAGTATTTCTGTAATTGAGGAGTCTTGCTCAGCAAATCCCACTTAGGGGACTAATGAAGTACCAGGAAGAGACAGACCATGCTCAATCCACAAAGCCAGGTTTTACTGAAATGTGACCTACTTTCTTATGCGATCGCCTGCCGAAAGAGTAATGTTGGCCGAGATAGGAGAAGACGATGATATCACGCTACGACGGAAACAGTACTCAGCAGAAATTGACGGAACAATGTAAATCAACTATAACAGAAGAAATAAAAACCTGGGGGGAAAGAAGCTGACTATGAAACCCCAGGAGCTTTCTACATGGGCCTGGACTCACCAAACTCTTTATTTTGTAATGGACTTCTGACATTTTTAGGAAGGGCTGTCCTGATGTGGGCTATAGAAGAGGGTTTCACATGCTTCTTCAAGAGGACCCACACTGTCCCAGTTGCTGAGTCCCACCACCAGATGCTAGTGGCAGCTATTTGGGGAACACTTAGGCACTACAAAAAAATGAGTGATTCCATTCTGGCTCACACCATATCCCTGATGTACCCCTTAAAGCATGTCACTGAGTTCATCACAGAAAATTGTTTCCCCTGTGCCTTCCACAACAAGGTTAGAGCTGTCCTTGGGGCCAGGGGAAGGGGGCAGGGAGTGAGAAGCACCAACTGGATAACCTCCTCTGACCCCCACTCCACCTTACCATAAGTAGATCCAAATCCTTCTAGAAAATTAGGAAGGCATATCCCCATATATCAGCGATATAAATAGAACTGCTTCAGCGCTCTGGTAGACGGTGACTCTCCAAGGTGGACTGGGAGGCAGCCTGGCCTTGGCTGGGCATCGTCCTCTAAATAGAAAGATGAACTTGTTCAGCCTTTCCAGAAGGAAAACTGCTGCCCAGCCTACAGTGCAACGTCCTTGTCTTCCATCTGGAGGAAGCACGGGTGACATATCATCTAGTAAGGGCACCTCTCTGTTTCCACCTCCAGGTCGAGGGGTGTGACCCTTACTTCTCAGCCTCAAGGGAGGGACACTCAACCCCCCAAAAAGACATGAGGGCGCTCAGCTCGGCCCACCGCACCCCGGACCGGAGCCGTCACCCCCCGAAATTCACTCCCTTCACAAGCCCCCAAGCGCGTTCTCTGGTGCGGACTGCTCCGGGGCCCTGGCTTTGTGCCCAGCGTTGTCAGAGCCACCGCCCTGAGCCTGTCCCCGGGAGCCCCGCGCCTCCTCCCACCGCTCCGCTCTCGCGCCCCGCGGCCAGTTGTCTGCCCCGAGACAGCTGCGCGCCCTCCCGCTGCCGGTGGCCCTCTCCGGTGGGGGTGGGGACCGACAGGGTCAGCCCTCCGGATCCGGGGCGCTCCGGGTAGCGGGGAGAAGTGATCGCTGGGGAGCTGGGGGAGGGGTCGCCTTCCTGCCCTACCCAGGACTCCGGGTGCGACCGCTCCTCTATCTCTCCAGCCCACCACCACTCCACCACTTGGACACGTCTCCCTCCTCCCTGGAGTCGCTCTAGAGGGTTTGGGGGTCTGAGTAAAGAACCCGAAGTAGGGATACAGTGTGGCGGCACCTTCCAGAGGCCCCGGGCGCAGGGTAGACCGGGGCGGGGCGGCCCGCGGACAGGTGCAGCCCCAGGCGCAGGCGCACTCGCGCCTCCCGGCGCAGGCGGTGAACCTCGCCCCACCCCAGCCCCTCCGGGGGGCAGCTGGGCCGGGTCGGGAGGGGCCCACCAGCCCGGGAGACACTCCATATACGGCCAGGCCCGCTTTACCTGGGCTCCGGCCAGGCCGCTCCTTCTTTGGTCAGCACAGGGGACCCGGGCGGGGGCCCAGGCCGCTAACCCGCCGGGGGAGGGGGCTCCAGTGCCCAACACCCAAATATGGCTCGAGAAGGGGAGCGACATTCCAGTGAGGCGGCTCGGGGGGAGAACCCGCGGGCTATATAAAACCTGAGCGTGGGGACCAGCGGCCACCGCAGCGGACAGCGCCGAGAGAAGCCTCGCTTCCCTCCCGCGGCGACCAGGGCCCCAGCCGGAGAGCAGCAGGTGTAGCCACCCGCCCAGGTAGACAAGAGTGGGATGGGGACAGGGAGACAGGGCACTGTCGAGGGGAGCCCAGAGAGCTTCCGGGAGCCCAGCCGACCCCATCTATTAGCCGCGACCCTGCCTCCTCGGTGCTGCTCGGTCCCCAGAGCCACATGCGCTTTAAAGAGGAGACAGGAAGCAGTCGACCTTTGGAAACGTCTCCAAATTTATTCCCTGCCCCTTTGGATTTCTCTACCTACACTTAGTATGTGTCCTGGTAGATGAACGTTTGACTTTAGTAGTCCCCAGGGCCAACCGAGCCAAAAGACAACAAGGGCCTCTCTTTCCGGCTTGCCATCAAGAGAAAGGGCGCCTAGGAATTTTTGAGTCTGGAATGTCGTGGGGTCTGGAAGAGGCTATTCCTCCCCGGATGGAACGCCCAAGCCCCACCCACGGCATGCCTTCCCACCCGTACAGTTTCCCAGCCCTTAGGGTTTGCCGGCCTTAACCCCCTTGGGGAGGCCACCGAGGTGCAGAGAGGGCGGGTGCCAGAGGGAAAGGGGAAGAAGAGTGTGCCCTGGGTTCCCAGGTGGAGAAATTCTGAGGTCTCTTCCCAGCGCAGTGCCCTGGCAGCGAAGCTCTGCCAGGTTGCTCGGATTGATCCCCTGGCGGGATGCACGAGGGCTGGGGGTGTCCTTAGGTCCCGGGGGAGGGAGGGCGCTCGGACCAGTGAGGCTTCCTGCCTGACACGCTGCGCATCTCCACGGCCTTGCTGATCTTGCAGAAACCCGACGCCGTTGCGGCCGCCACCATGGTCGCTCATTCCTCCGACGGGCTGTCCGCCACCGCTCCTGTGACCGGCGGAGATGTGCTGGTCGATGCTCGTGTTTCTATTGAAGAGAAGCCCCCTCGCTCCCTGGACTCCACTCAACAGTCTACTGAGGAGGAGCGCGTGCAATTGCCCACTGTGGATGCTTTCCGCCGCGCCATTCCACCCCATTGCTTCGAACGCGATCTGACCAGATCCCTCCGATATCTGGTGCAAGACTTTGCCGCTCTGGCTTTTCTCTACTTTGCTCTGCCTGTGTTCGAATACTTTGGACTGGTGGGCTATCTGGCTTGGAACGTGCTGATGGGCGTCTTCGGATTCGCTTTGTTCGTGGTCGGCCACGATTGTCTGCATGGATCCTTCTCCGATAACCAAGTGCTGAACGATATTATTGGCCACATCGCTTTCTCCCCTCTGTTCTCCCCCTATTTCCCTTGGCAGAAGTCCCACAAACTGCACCACGCTTTCACCAACCATATTGACAAGGATCACGGACACGTGTGGATTCAAGACAAAGACTATGAAAAGATGCCTACATGGAAGAAGCTGTTCAACCCTATGCCCTTCTCCGGATGGCTGAAATGGTTCCCCGTGTACACTCTGTTCGGATTCTGCGATGGATCCCATTTCTGGCCTTACTCCTCTCTGTTCGTGCGCGATTCCGAGCGCGTCCAATGCGTGATTTCCGCTACTTGCTGTGTGGCCTGTGCCTATGTGGCTCTGGCTATTGCCGGCTCCTACTCCAACTGGTTCTGGTACTACTGGGTGCCTCTGTCCTTCTTCGGATGCATGCTGGTGATTGTCACCTATCTGCAACACGCTGATGAAGTGGCTGAGGTGTACGAAGCTGATGAGTGGAGTTTCGTGCGTGGACAAACCCAGACTATCGACCGCTTCTATGGATTTGGACTGGATGAGACCATGCACCATATTACTGACGGACACGTGGCCCATCACTTCTTCAATAAGATTCCTCATTACCATCTGATCGAAGCTACTGAAGGTGTGAAGAAGGTGTTGGAGCCTCTGTTCGAGACTCAGTACGGATACAAGTACCAAGTGAACTACGACTTCTTCGTCCGCTTCCTGTGGTTCAACCTGAAGCTGGACTATCTGGTGCATAAGACTAAAGGTATCCTGCAATTCCGCACAACTCTGGAGGAGAAGGCGAAGGCCAAGTAACGCGCCAAGCGGGCTCCTGTCAAGCAGACCCTGAACTTCGACCTGCTGAAGCTGGCTGGCGACGTGGAGAGCAACCCTGGCCCCATGGTCCGTCCCAAGCACCAGCCCGGCGGGCTATGCCTCCTGCTGCTGCTGCTCTGCCAGTTCATGGAGGACCGCAGCGCCCAGGCTGGGAATTGCTGGCTCCGCCAAGCAAAAAACGGCCGCTGCCAGGTCCTGTATAAAACCGAACTGAGCAAGGAGGAGTGCTGCAGCACCGGCCGCCTGAGCACCTCCTGGACTGAGGAGGACGTAAATGACAACACACTTTTCAAGTGGATGATTTTCAATGGAGGTGCCCCCAGCTGCATCCCATGTAAAGAAACGTGCGAGAACGTGGACTGTGGGCCCGGGAAAAAATGCCGAATGAACAAGAAGAACAAACCCCGCTGCGTCTGCGCCCCGGATTGTTCTAACATCACCTGGAAAGGCCCAGTCTGTGGGCTGGATGGGAAAACCTACCGCAACGAATGTGCTCTCCTCAAGGCCAGATGTAAAGAGCAGCCGGAACTGGAAGTCCAGTACCAAGGCAAATGTAAAAAGACCTGTCGGGATGTTTTCTGTCCAGGCAGCTCCACATGTGTGGTGGACCAGACTAATAATGCCTACTGTGTGACATGTAACCGCATTTGCCCAGAGCCCACCTCCTCAGGACAGTATCTCTGTGGGAATGATGGAGTGACCTACTCCAGTGCCTGTCACCTGAGAAAGGCTACCTGCCTACTGGGCAGATCTATTGGATTGGCCTATGAGGGAAAGTGTATCAAAGCAAAGTCCTGTGAAGACATCCAGTGCACTGGTGGAAAAAAGTGCTTATGGGATTTCAAGGCTGGCAGAGGCCGCTGTTCCCTCTGCGATGAGCTGTGCCCTGAGAGTAAGTCTGAGGAGCCTGTCTGTGCCAGTGACAATGCCACCTACGCCAGTGAGTGTGCCATGAAGGAAGCGGCCTGTTCCTCAGGTGTGCTGCTGGAAGTAAAGCACTCTGGATCTTGCAACTCCATTTCAGAAGACACCGAGGAGGAGGAGGAAGATGAAGACCAGGACTACAGCTTTCCTATATCTTCTATTCTAGAGTGGTAAACCCCATTCCAACAGCTGCGACTTCTCCTCAGGACGACGAATCTGCTCAAGTGCAGTGGCTGACCTCGAGCCACCAAGCGGCAACTGCTCTCCCACCGCCTGCGTTGCTGCTGCCCAAACCGACACAATGTTTATAACGTGTACATACATTAACTTATTTACCTCATTTTGTTATTTTTAAAACGAAGCCCTGTGGAAGGAAATGGAAAACTTGAAGCATTAAACTCAGCCATTCTGTTATGCTGCGTAAAATTGTCTGCTGTGTTTATTTGCGGGGGTGAGGAGCGGCGGGGGTGGGGAGCGGCGGGGGTCGGGAGGAAGAGGGACCCCCAGCTTTCTACCTCAGCGTCACTTTTCACAATACTCTGAACGAATGCACTCTTTTCAAAACCATGATTAAATCTCCCCGTACTCATGATTTTCCCCTCTGGCTCAGATCGGTTCCCTGTACTGGCAGGCCAGCCGAGGCCCGACTTGACCCTGGGGCGTGTCCCCCTTGTCCCTGTGCTGGGGCTGCAGCCAGCGATGGGGCAGCAGCGCCGGCGTCTGGGTCTTGACGCCGGCAGGGCGCTGACCGTGGTGCTGGGACCGAGGGCCGCCCTGCAAAGCGTATTCCTGCTCCTCCTACGCGTTCCTTATTGAATCTCGAAATCTAAATGAAAGGGCTACATCCGCTTTTAAAGTACCGGTTCTCATTTACAAAGTGAAGATAACCACTTCTATTCCGGACTGAGACGGCCTTTGCTTCAAGCCGGGTGTTGATAGTACTTTAATTAAACGCGGTGGCGGCCGCGAATTCACTAGTGATTTCCCCGGGAGATCTAATTCTAGCTAGATAACTTCGTATAGCATACATTATACGAAGTTATAGCTTCTGATGGAATTAGAACTTGGCAAAACAATACTGAGAATGAAGTGTATGTGGAACAGAGGCTGCTGATCTCGTTCTTCAGGCTATGAAACTGACACATTTGGAAACCACAGTACTTAGAACCACAAAGTGGGAATCAAGAGAAAAACAATGATCCCACGAGAGATCTATAGATCTATAGATCATGAGTGGGAGGAATGAGCTGGCCCTTAATTTGGTTTTGCTTGTTTAAATTATGATATCCAACTATGAAACATTATCATAAAGCAATAGTAAAGAGCCTTCAGTAAAGAGCAGGCATTTATCTAATCCCACCCCACCCCCACCCCCGTAGCTCCAATCCTTCCATTCAAAATGTAGGTACTCTGTTCTCACCCTTCTTAACAAAGTATGACAGGAAAAACTTCCATTTTAGTGGACATCTTTATTGTTTAATAGATCATCAATTTCGGGGTGGGCGAAGAACTCCAGCATGAGATCCCCGCGCTGGAGGATCATCCAGCCGGCGTCCCGGAAAACGATTCCGAAGCCCAACCTTTCATAGAAGGCGGCGGTGGAATCGAAATCTCGTGATGGCAGGTTGGGCGTCGCTTGGTCGGTCATTTCGAACCCCAGAGTCCCGCTCAGAAGAACTCGTCAAGAAGGCGATAGAAGGCGATGCGCTGCGAATCGGGAGCGGCGATACCGTAAAGCACGAGGAAGCGGTCAGCCCATTCGCCGCCAAGCTCTTCAGCAATATCACGGGTAGCCAACGCTATGTCCTGATAGCGGTCCGCCACACCCAGCCGGCCACAGTCGATGAATCCAGAAAAGCGGCCATTTTCCACCATGATATTCGGCAAGCAGGCATCGCCATGGGTCACGACGAGATCCTCGCCGTCGGGCATGCGCGCCTTGAGCCTGGCGAACAGTTCGGCTGGCGCGAGCCCCTGATGCTCTTCGTCCAGATCATCCTGATCGACAAGACCGGCTTCCATCCGAGTACGTGCTCGCTCGATGCGATGTTTCGCTTGGTGGTCGAATGGGCAGGTAGCCGGATCAAGCGTATGCAGCCGCCGCATTGCATCAGCCATGATGGATACTTTCTCGGCAGGAGCAAGGTGAGATGACAGGAGATCCTGCCCCGGCACTTCGCCCAATAGCAGCCAGTCCCTTCCCGCTTCAGTGACAACGTCGAGCACAGCTGCGCAAGGAACGCCCGTCGTGGCCAGCCACGATAGCCGCGCTGCCTCGTCCTGCAGTTCATTCAGGGCACCGGACAGGTCGGTCTTGACAAAAAGAACCGGGCGCCCCTGCGCTGACAGCCGGAACACGGCGGCATCAGAGCAGCCGATTGTCTGTTGTGCCCAGTCATAGCCGAATAGCCTCTCCACCCAAGCGGCCGGAGAACCTGCGTGCAATCCATCTTGTTCAATCATGCGAAACGATCGGGCAAACGTGCGCGCCAGGTCGCATATCGTCGGTATGGAGCCGGGGGTGGTGACGTGGGTCTGGACCATCCCGGAGGTAAGTTGCAGCAGGGCGTCCCGGCAGCCGGCGGGCGATTGGTCGTAATCCAGGATAAAGACGTGCATGGAACGGAGGCGTTTGGCCAAGACGTCCAAGGCCCAGGCAAACACGTTATACAGGTCGCCGTTGGGGGCCAGCAACTCGGGGGCCCGAAACAGGGTAAATAACGTGTCCCCGATATGGGGTCGTGGGCCCGCGTTGCTCTGGGGCTCGGCACCCTGGGGCGGCACGGCCGTCCCCGAAAGCTGTCCCCAGTCCTCCCGCCACGACCCGCCGCACTGCAGATACCGCACCGTATTGGCAAGTAGCCCGTAAACGCGGCGAATCGCAGCCAGCATAGCCAGGTCCAGCCGCTCGCCGGGGCGCTGGCGTTTGGCCAGGCGGTCGATGTGTCTGTCCTCCGGAAGGGCCCCAAGCACGATGTTGGTGCCGGGCAAGGTCGGCGGGATGAGGGCCACGAACGCCAGCACGGCCTGGGGGGTCATGCTGCCCATAAGGTACCGCGCGGCCGGGTAGCACAGGAGGGCGGCGATGGGATGGCGGTCGAAGATGAGGGTGAGGCCCGGGGGCGGGGCATGTGAGCTCCCAGCCTCCCCCCCGATATGAGGAGCCAGAACGGCGTCGGTCACGGCATAAGGCATGCCCATTGTTATCTGGGCGCTTGTCATTACCACCGCCGCGTCCCCGGCCGATATCTCACCCTGGTCGAGGCGGTGTTGTGTGGTGTAGATGTTCGCGATTGTCTCGGAAGCCCCCAGCACCCGCCAGTAAGTCATCGGCTCGGGTACGTAGACGATATCGTCGCGCGAACCCAGGGCCACCAGCAGTTGCGTGGTGGTGGTTTTCCCCATCCCGTGGGGACCGTCTATATAAACCCGCAGTAGCGTGGGCATTTTCTGCTCCGGGCGGACTTCCGTGGCTTCTTGCTGCCGGCGAGGGCGCAACGCCGTACGTCGGTTGCTATGGCCGCGAGAACGCGCAGCCTGTTCGAACGCAGACGCGTGTTGATGGCCGGGGTACGAAGCCATACGCGCTTTTACAAGGCGCTGGCCGAAGAGGTGCGGGAGTTTCACGCCACCAAGATCGGTCGAAAGGCCCGGAGATGAGGAAGAGGAGAACAGCGCGGCAGACGTGCGCTTTTGAAGCGTGCAGAATGCCGGGCCTCCGGAGGACCTTCGGGCGCCCGCCCCGCCCCTGAGCCCGCCCCTGAGCCCGCCCCCGGACCCACCCCTTCCCAGCCTCTGAGCCCAGAAAGCGAAGGAGCAAAGCTGCTATTGGCCGCTGCCCCAAAGGCCTACCCGCTTCCATTGCTCAGCGGTGCTGTCCATCTGCACGAGACTAGTGAGACGTGCTACTTCCATTTGTCACGTCCTGCACGACGCGAGCTGCGGGGCGGGGGGGAACTTCCTGACTAGGGGAGGAGTAGAAGGTGGCGCGAAGGGGCCACCAAAGAACGGAGCCGGTTGGCGCCTACCGGTGGATGTGGAATGTGTGCGAGGCCAGAGGCCACTTGTGTAGCGCCAAGTGCCCAGCGGGGCTGCTAAAGCGCATGCTCCAGACTGCCTTGGGAAAAGCGCCTCCCCTACCCGGTAGAATTCATAACTTCGTATAGCATACATTATACGAAGTTATGGATCCGGTACCGAGCTTCCAATCGAATTCCCGCGGCCGGGCCGGCCGCGGCCGCTTCCCGAGGCTGAGTTAGTTGGTCCAGCCAGTGATTGAGTTGCGTGCGGAGGGCTTCTTATCTTAGTTTTATAGGCTACACTGTTAACACTCAGGCTGTTTTCTACCGTTTAGTCAAAATATAGTCACCTTGCCTGCTTCACCTGTCCATCAGAGAATGGCCTCATTAATTGACTCTCTAGTATGAAGTCAAAGTAGCTTTGGTGGCCCTAAATGGACAAGTATCAAGAGACTGGGTGAATTGAGGAGCTTGAGACTGTCACCTCAGATCGAAAAGACTGAAAAATCACCTCAGATCAAAAAGACTGAAAAATCTTCAGTCTGGAAAGGGGACTCAAAACCATAATTAGAGTATTCTGGTAGAATCCTTTTCTCCACTGTTATTCATACAGTTAAGGTGAATAACTAAAAGTAATTGTGAGCTGAGGAGTAAGATACAACACACAAGGAATCAGTTAACAGAGTCTCGAGTGAAATTATAAATGGAAAGAATTATGACTTGAATCATAACTCTGAGGCCCCATTTTCCCTAACAACTTTTGTCCCAATAAACGTGGGTATTTGTTTGGGAGAAACTATCATATACATGATTACCCAGTAAACAGACTGTTTACTAAGTGGGTTTAATTTTAGAAATTGCGCGCTGCAATCTGGTATTAACCATACAACTACCTACCTATAGGGTCAGCCCAGCCTGAACTATCCCATTGGGGTCTTTATTAAGGCTCAAGAAACGGCCATAGCTTCTTCCTTTAAAATGAGTGTTTATTTCTATGAGCTTTAAAGAAAAAAACAGATAATTTCCCTCAACCTACTGAAGAGGAAGGGATTCAGGAAGAAATAAACACAACAATGCCATTCACTTCAATGCATGTTTAAACAGGCCGCGGGAATTCGATTATCGAATTCTACCGGGTAGGGGAGGCGCTTTTCCCAAGGCAGTCTGGAGCATGCGCTTTAGCAGCCCCGCTGGGTCACTTGGCGCTACACAAGTGGCCTCTGGCCTCGCACACATTCCACATCCACCGGTAGGCGCCAACCGGCTCCGTTCTTTGGTGGCCCCTTCGCGCCACCTTCTACTCCTCCCCTAGTCAGGAAGTTCCCCCCCGCCCCGCAGCTCGCGTCGTGCAGGACGTGACAAATGGAAGTAGCACGTCTCACTAGTCTCGTGCAGATGGACAGCACCGCTGAGCAATGGAAGCGGGTAGGCCTTTGGGGCAGCGGCCAATAGCAGCTTTGCTCCTTCGCTTTCTGGGCTCAGAGGCTGGGAAGGGGTGGGTCCGGGGGCGGGCTCAGGGGCGGGCTCAGGGGCGGGGCGGGCGCCCGAAGGTCCTCCGGAGGCCCGGCATTCTGCACGCTTCAAAAGCGCACGTCTGCCGCGCTGTTCTCCTCTTCCTCATCTCCGGGCCTTTCGACCTGCAGGTCCTCGCCATGGATCCTGATGATGTTGTTGATTCTTCTAAATCTTTTGTGATGGAAAACTTTTCTTCGTACCACGGGACTAAACCTGGTTATGTAGATTCCATTCAAAAAGGTATACAAAAGCCAAAATCTGGTACACAAGGAAATTATGACGATGATTGGAAAGGGTTTTATAGTACCGACAATAAATACGACGCTGCGGGATACTCTGTAGATAATGAAAACCCGCTCTCTGGAAAAGCTGGAGGCGTGGTCAAAGTGACGTATCCAGGACTGACGAAGGTTCTCGCACTAAAAGTGGATAATGCCGAAACTATTAAGAAAGAGTTAGGTTTAAGTCTCACTGAACCGTTGATGGAGCAAGTCGGAACGGAAGAGTTTATCAAAAGGTTCGGTGATGGTGCTTCGCGTGTAGTGCTCAGCCTTCCCTTCGCTGAGGGGAGTTCTAGCGTTGAATATATTAATAACTGGGAACAGGCGAAAGCGTTAAGCGTAGAACTTGAGATTAATTTTGAAACCCGTGGAAAACGTGGCCAAGATGCGATGTATGAGTATATGGCTCAAGCCTGTGCAGGAAATCGTGTCAGGCGATCTCTTTGTGAAGGAACCTTACTTCTGTGGTGTGACATAATTGGACAAACTACCTACAGAGATTTAAAGCTCTAAGGTAAATATAAAATTTTTAAGTGTATAATGTGTTAAACTACTGATTCTAATTGTTTGTGTATTTTAGATTCCAACCTATGGAACTGATGAATGGGAGCAGTGGTGGAATGCAGATCCTAGAGCTCGCTGATCAGCCTCGACTGTGCCTTCTAGTTGCCAGCCATCTATTGTTTGCCCCTCCCCCGTGCCTTCCTTGACCCTGGAAGGTGCCACTCCCACTGTCCTTTCCTAATAAAATGAGGAAATTGCATCGCATTGTCTGAGTAGGTGTCATTCTATTCTGGGGGGTGGGGTGGGGCAGGACAGCAAGGGGGAGGATTGGGAAGACAATAGCAGGCATGCTGGGGATGCGGTGGGCTCTATGGCTTCTGAGGCGGAAAGAACCAGCTGGGGCTCGAGGGGGGGCCCGGTACCCAATTCGCC


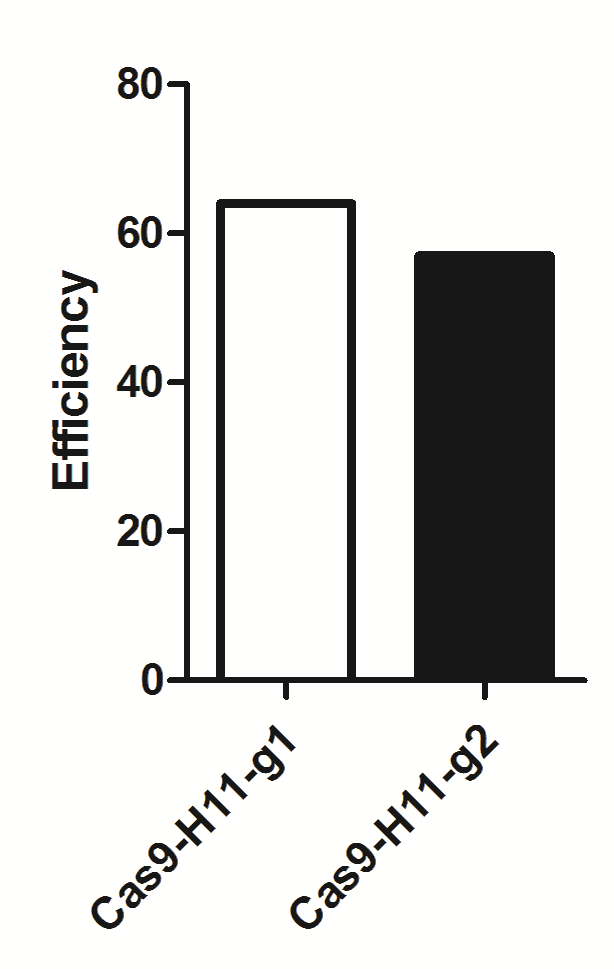


**Figure S1. Efficiency of sgRNA Cas9-H11-g1 and Cas9-H11-g2 based on sequence results.** Y axis stands for efficiency of sgRNA calculated using T7EI assay.. Cas9-H11-g1 targeting efficiency is 64% and Cas9-H11-g2 targeting efficiency is 57%.


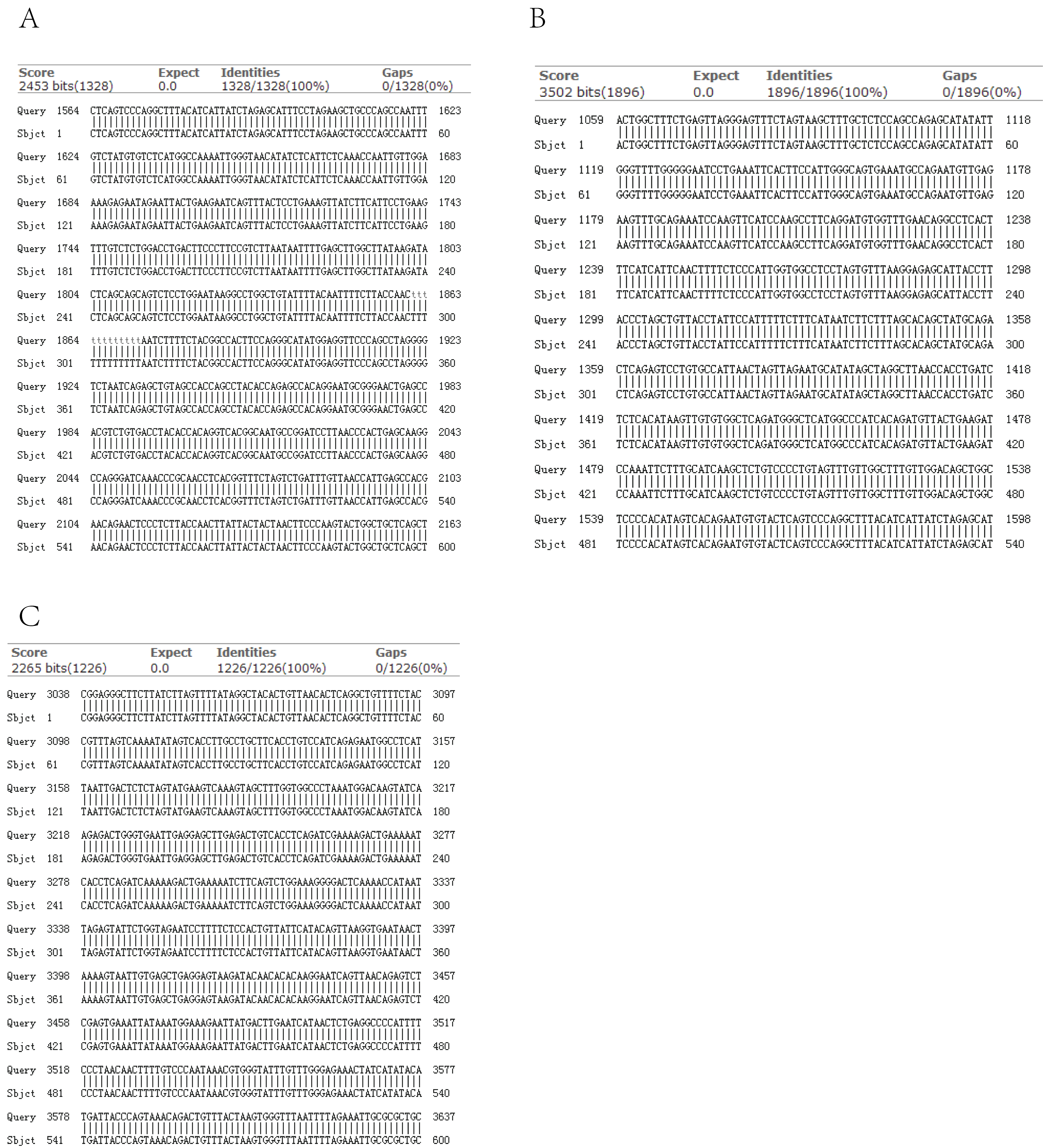


**Figure S2. Blast results comparing PCR sequences with genomic junction sequences for screening positive clones.** (A) Blast results for primer set P1. (B) Blast results for primerset P2. (C) Blast results for primerset P3. P1 and P2 are primer sets for amplifying the 5’ junction and P3 is a primer set for amplifying the 3’ junction.


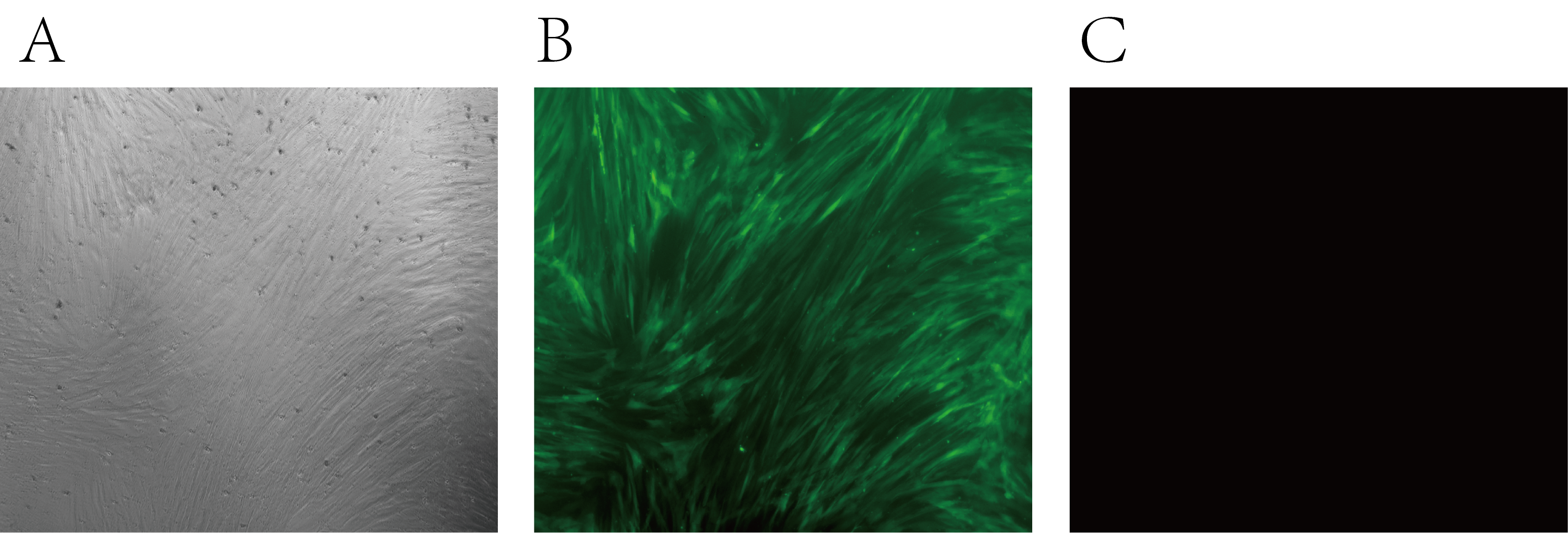


**Figure S3. Examples of positive fibroblast cell clones with GFP expression under a fluorescence stereomicroscope.** (A) Cells under white light. (B) Cells under green fluorescence. (C) Cells under red fluorescence.


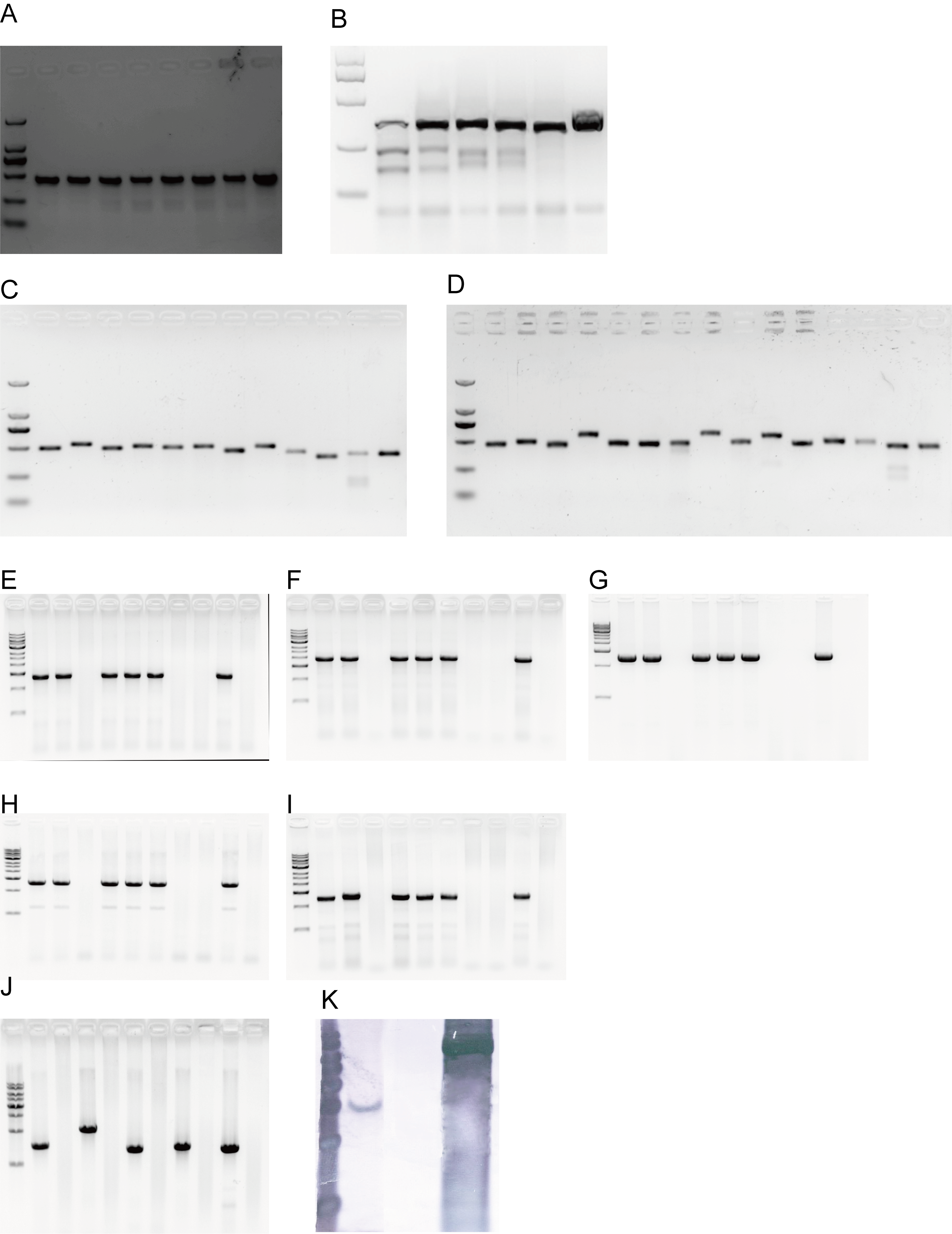


**Figure S4. Full length gels and blots.** (A) Full-length gels results of Figure1 C. (B) Full-length gels results of Figure 1D. (C) Full-length gels results of Figure 2A. (D) Full-length gels results of Figure 2B. (E) Full-length gels results of P1 that in Figure 3C. (F) Full-length gels results of P2 that in Figure 3C. (G) Full-length gels results of P3 that in Figure 3C. (H) Full-length gels results of GFP that in Figure 3C. (I) Full-length gels results of Neo that in Figure 3C. (J) Full-length gels results of Figure 4D. (K) Full-length Blots results of Figure 4F.

**Table S1 PCR primers for amplifying predicted off-target sites for sgRNA Cas9-H11-g1 (g1) and Cas9-H11-g2 (g2)**

| Primer Name | Sequence (5’ to 3’ ) | PCR product | Product size |
| --- | --- | --- | --- |
| g1P1_F | AAAGGAGGCGGAAGGGTAG | g1P1 | 494 bp |
| g1P1_R | GAGGAAGCTGTGAAGGTGGAG |
| g1P2_F | GGGCAGCTAAAGAAATTGTGC | g1P2 | 541 bp |
| g1P2_R | GACTGCATGTGTGTCCCCTC |
| g1P3_F | GCAACGCGGGATCTAAACTG | g1P3 | 496 bp |
| g1P3_R | AACCTCTTAGCAGACACGACAAC |
| g1P4_F | CCAGATTAGAGGCAAGTTCGG | g1P4 | 513 bp |
| g1P4_R | TATGCTGGGCTGTGGATGG |
| g1P5_F | CTTCATAGCAATTTCCTTATCC | g1P5 | 504 bp |
| g1P5_R | AGGGTTAGGGTAGAATTGGAGC |
| g1P6_F | AGCAACATTACATTAGGAAACCG | g1P6 | 510 bp |
| g1P6_R | TGACTTGTGGTAACTTAGGACTGTG |
| g1P7_F | TCCAGACCTTAACTCTCCTGAAC | g1P7 | 466 bp |
| g1P7_R | GTCCTGGTCCTGCCACTTG |
| g1P8_F | AGCTGTGTTCAGGTCTCGTCC | g1P8 | 524 bp |
| g1P8_R | GACCCCTATGCTTGGGAACC |
| g1P9_F | AAAAGTTGTATTTCTTCCCTCTCA | g1P9 | 457 bp |
| g1P9_R | CTCCCCCATCACTTTCTCTG |
| g1P10_F | AAAACCCATGTGACAACCAAGA | g1P10 | 416 |
| g1P10_R | TATAAGCAAATCCTTTCCGAGG |
| g2P1_F | AGGACAGCAGTGGTGGGATG | g2P1 | 456 bp |
| g2P1_R | AAGTGTGAGTCTGTGAGCCGC |
| g2P2_F | AGGATGGACAGGTGTGTTTGG | g2P2 | 487 bp |
| g2P2_R | GGCTTGAAAGAGCATGGCAC |
| g2P3_F | CCTGCTTAGGTTCCATTTGCC | g2P3 | 446 bp |
| g2P3_R | GGCATACATGATGGTGGCTCC |
| g2P4_F | CACCAAGTGGAAGTGGGCTC | g2P4 | 555 bp |
| g2P4_R | TGTTGGATTGCTGCGTCTTC |
| g2P5_F | ATCCTGGATGTGAGCATTGTGT | g2P5 | 468 bp |
| g2P5_R | GGAGCCGACAGACATCAACC |
| g2P6_F | GGTGGGTTAGTCATTTCAGGTG | g2P6 | 463 bp |
| g2P6_R | GGAAACCCGCAGTAAAGGC |
| g2P7_F | CACTGCACAGAGGACTTATCACAC | g2P7 | 470 bp |
| g2P7_R | GATTTTACCTTGATGGCGGC |
| g2P8_F | GACAGGTGTAGAGGGAGGCG | g2P8 | 584 bp |
| g2P8_R | GAGGCAGGGATGGAAGCAG |
| g2P9_F | TTATCCTGGGCTCCTTTCTCTC | g2P9 | 483 |
| g2P9_R | CAGGCTTGCATTTTGTGTGAG |
| g2P10_F | CGAGGATTTTCAGCCCCAG | g2P10 | 554 bp |
| g2P10_R | GTCCCATCTGTGCCCGTTC |
| g2P11_F | ATGGCGACATGGTTAGAGCAC | g2P11 | 535 bp |
| g2P11_R | CTTAACCCACTGAGCGAGGC |
| g2P12_F | CCTCGGGCTCTCCCTATGTG | g2P12 | 486 bp |
| g2P12_R | CTACCCATCCCCCAAAGGC |
| g2P13_F | TCCCAGGCTAGGAGTTGAATC | g2P13 | 482 bp |
| g2P13_R | TCATCAATGCCAGCTTGTCAC |

g1: sgRNA Cas9-H11-g1; g2: sgRNA Cas9-H11-g2; P: primer; F: forward primer; R: reverse primer.

**Table S2 Primers used in this study.**

| Primer Name | Sequence (5’ to 3’) | Sequence Site | Product Size |
| --- | --- | --- | --- |
| pH11-up | GATGGATTGGGATTCAGGTCTC | *H11* locus | 447 bp |
| pH11-dn | AAGCTCCTCAATTCACCCAGTC |
| H11-P1-F | CTCAGTCCCAGGCTTTACATC | 5’- junction (P1) | 1349 bp |
| H11-P1-R | CCAACATTACTCTTTCGGCAG |
| H11-P2-F | ACTGGCTTTCTGAGTTAGGG | 5’- junction (P2) | 1896 bp |
| H11-P2-R | GTTTCCGTCGTAGCGTGATA |
| H11-P3-F | CGGAGGGCTTCTTATCTTAG | 3’- junction (P3) | 1226 bp |
| H11-P3-R | GTGTGGAGCTGTTTAGGGAC |
| GFP-F | AGCCCCTTGTTGAATACGCT | GFP | 1295 bp |
| GFP-R | GGCACCAAAATCAACGGGAC |
| Neo-F | GCCGCATTACCCTGTTATCC | Neo | 1261 bp |
| Neo-R | GGGGACGTGGTTTTCCTTTGA |
| Southern-F | TCTTGTAGTTGCCGTCGTC | Southern | 523bp |
| Southern-R | GGATAGCGGTTTGACTCAC |

P: primer; F: forward; R: reverse; GFP: green fluorescence protein; Neo: neomycin; Southern: Southern Blot.
